# Novel Potential Therapeutic Targets of PTPN Families for Lung Cancer

**DOI:** 10.3390/jpm12121947

**Published:** 2022-11-23

**Authors:** Chin-Chou Wang, Wan-Jou Shen, Gangga Anuraga, Hoang Dang Khoa Ta, Do Thi Minh Xuan, Sih-Tong Chen, Chiu-Fan Shen, Jia-Zhen Jiang, Zhengda Sun, Chih-Yang Wang, Wei-Jan Wang

**Affiliations:** 1 Divisions of Pulmonary & Critical Care Medicine, Department of Internal Medicine, Kaohsiung Chang Gung Memorial Hospital, Chang Gung University College of Medicine, Kaohsiung 83301, Taiwan; 2 Department of Respiratory Therapy, Kaohsiung Chang Gung Memorial Hospital, Chang Gung University College of Medicine, Kaohsiung 83301, Taiwan; 3 Department of Respiratory Care, Chang Gung University of Science and Technology, Chiayi 613016, Taiwan; 4 Department of Biological Science and Technology, China Medical University, Taichung 40676, Taiwan; 5 Graduate Institute of Cancer Biology and Drug Discovery, College of Medical Science and Technology, Taipei Medical University, Taipei 11031, Taiwan; 6 Ph.D. Program for Cancer Molecular Biology and Drug Discovery, College of Medical Science and Technology, Taipei Medical University and Academia Sinica, Taipei 11031, Taiwan; 7 Department of Statistics, Faculty of Science and Technology, Universitas PGRI Adi Buana, Surabaya 60234, Indonesia; 8 Emergency Department, Huashan Hospital North, Fudan University, Shanghai 201508, China; 9 Kaiser Permanente, Northern California Regional Laboratories, The Permanente Medical Group, 1725 Eastshore Hwy, Berkeley, CA 94710, USA; 10 TMU Research Center of Cancer Translational Medicine, Taipei Medical University, Taipei 11031, Taiwan; 11 Research Center for Cancer Biology, China Medical University, Taichung 40676, Taiwan

**Keywords:** PTPN family genes, lung cancer, prognosis, bioinformatics, big data analysis

## Abstract

Despite the treatment of lung adenocarcinoma (LUAD) having partially improved in recent years, LUAD patients still have poor prognosis rates. Therefore, it is especially important to explore effective biomarkers and exploit novel therapeutic developments. High-throughput technologies are widely used as systematic approaches to explore differences in expressions of thousands of genes for both biological and genomic systems. Recently, using big data analyses in biomedicine research by integrating several high-throughput databases and tools, including The Cancer Genome Atlas (TCGA), cBioportal, Oncomine, and Kaplan–Meier plotter, is an important strategy to identify novel biomarkers for cancer therapy. Here, we used two different comprehensive bioinformatics analysis and revealed protein tyrosine phosphatase non-receptor type (PTPN) family genes, especially PTPN1 and PTPN22, were downregulated in lung cancer tissue in comparison with normal samples. The survival curves indicated that LUAD patients with high transcription levels of PTPN5 were significantly associated with a good prognosis. Meanwhile, Gene Ontology (GO) and MetaCore analyses indicated that co-expression of the PTPN1, PTPN5, and PTPN21 genes was significantly enriched in cancer development-related pathways, including GTPase activity, regulation of small GTPase-mediated signal transduction, response to mechanical stimuli, vasculogenesis, organ morphogenesis, regulation of stress fiber assembly, mitogen-activated protein kinase (MAPK) cascade, cell migration, and angiogenesis. Collectively, this study revealed that PTPN family members are both significant prognostic biomarkers for lung cancer progression and promising clinical therapeutic targets, which provide new targets for treating LUAD patients.

## 1. Introduction

Lung cancer is the leading cause of cancer deaths worldwide, accounting for 18.0% of total cancer deaths [1]. Lung cancer is composed of small-cell lung carcinoma (SCLC) and non-SCLC (NSCLC). NSCLC comprises around 85% of lung cancer cases and it is further classified into three subtypes, squamous-cell carcinoma, adenocarcinoma, and large-cell carcinoma [2]. Lung adenocarcinoma (LUAD) is the main subtype of NSCLC, accounting for 40% of all cases of lung cancer. Despite the treatment of LUAD having partially improved in recent years, LUAD patients still have a relatively poor prognosis, and the 5-year relative survival rate is less than 21% [3,4]. Therefore, it is especially important to explore effective biomarkers and exploit novel therapeutic developments [5,6,7,8,9].

The tyrosine phosphorylation of proteins plays a key role in controlling several signaling pathways involved in cell proliferation, apoptosis, migration, and invasion [10]. The process of signal transduction is coordinately mediated by protein tyrosine phosphatases (PTPs) and protein tyrosine kinases (PTKs), and it is orchestrated by a cascade of molecular events involving protein phosphorylation by PTKs and de-phosphorylation by PTPs [11]. Notably, published research suggests that PTPs may have essential roles in tumor invasion and metastasis [12]. The PTP family comprises 107 members that can be divided into four separate classes according to differences in amino acid sequence of the catalytic domain. The protein tyrosine phosphatase non-receptor type (PTPN) family is part of class I cysteine-based PTPs [13]. There are 17 different members involved in the PTPN family in humans, and accumulating evidence suggests that PTPN family members have critical roles in the progression of a variety of human cancers. For instance, it was demonstrated that PTPN3 acts as a suppressor in tumorigenesis, and it could modulate the transforming growth factor (TGF)-β signaling in liver cancer [14]. Published research has shown that mutations and genetic variants of certain genes in the PTPN2 pathway are related to the risk and survival of lung cancer [15]. The loss of PTPN4 by activation of signal transduction and activator of transcription 3 (STAT3) might cause tumorigenesis of colorectal cancer [16]. In addition, the current study revealed that PTPN11 has an oncogenic function in breast cancer by regulating the phosphatidylinositol 3-kinase (PI3K)/AKT/glycogen synthase kinase 3β (GSK3β) signaling pathway. [17] However, relationships between PTPN family members and LUAD have not been extensively studied.

In the present study, we utilized large-scale bioinformatics databases to identify the expression status of different PTPN members in LUAD patients [18,19,20,21,22,23]. The main purpose of this study was to determine expression patterns and molecular mechanisms of PTPN for LUAD, define predictive biomarkers, and develop new therapeutic targets.

## 2. Materials and Methods

### 2.1. UALCAN Analysis

The Cancer Genome Atlas (TCGA) level 3 RNA-sequencing (RNA-Seq) together with clinical data acquired from around 30 different types of cancer are combined in UALCAN [24,25,26,27,28]. Expression values of more than 25,000 genes within this database were calculated using the RNA-Seq by Expectation Maximization (RSEM) tool. Transcripts per million (TPM) measurements were applied to determine whether there were statistically significant differences in gene expression levels among groups. This platform was also employed to extract TCGA data, which included 515 primary LUAD samples and 59 normal samples. In our study, we examined messenger (m)RNA levels of five PTPN family genes in LUAD and their relationships to clinicopathological characteristics and tumor progression presented at different stages.

### 2.2. Cancer Cell Line Encyclopedia (CCLE) Analysis

The CCLE project was employed to further examine expression level patterns of all PTPN members on a broader scale. This web-based application gave users access to more than 1000 human cancer cell lines and 130 different datasets that are both genetically and pharmacologically characterized [29,30,31,32]. Additionally, independent LUAD cancer cell lines were subjected to the integrated RNA-Seq Aligned Reads method to plot the expression of each PTPN gene.

### 2.3. Functional Enrichment Analysis of PTPN Target Genes

The InteractiVenn tool was selected to generate a one-way Venn diagram that presents the overlap and number of genes linked with the expression of PTPN target genes across the two given datasets obtained from TCGA database (available at the cBioPortal platform), and together with the MetaCore platform was employed to map the intersection between these two sets of data in terms of related pathways and involved networks. A *p* value of <0.05 was as previously described [33,34,35,36,37].

### 2.4. Kaplan–Meier (KM) Overall Survival (OS) Analysis

The KM database is an integrated online database available at https://kmplot.com/ (accessed on 14 April 2021) which prominently features in assessing target genes in survivors of more than 20 different types of cancer. This tool was subsequently leveraged to expand on LUAD prognosis-related issues, including OS, first progression (FP), and post-progression survival (PPS) [31]. The independent prognostic values of PTPN target genes on patients diagnosed with LUAD as mentioned above were represented as two comparative KM survival curves, one for LUAD patients and one for the healthy population, by concurrently integrating messenger (m)RNA expression levels and clinical data obtained from the target genes. Comparisons of the two patient cohorts were performed with 95% confidence intervals of hazard ratios and a fixed log-rank *p* value [38,39,40].

## 3. Results

### 3.1. Expression Patterns of PTPN Family Members in LUAD Patients

**Figure 1:**
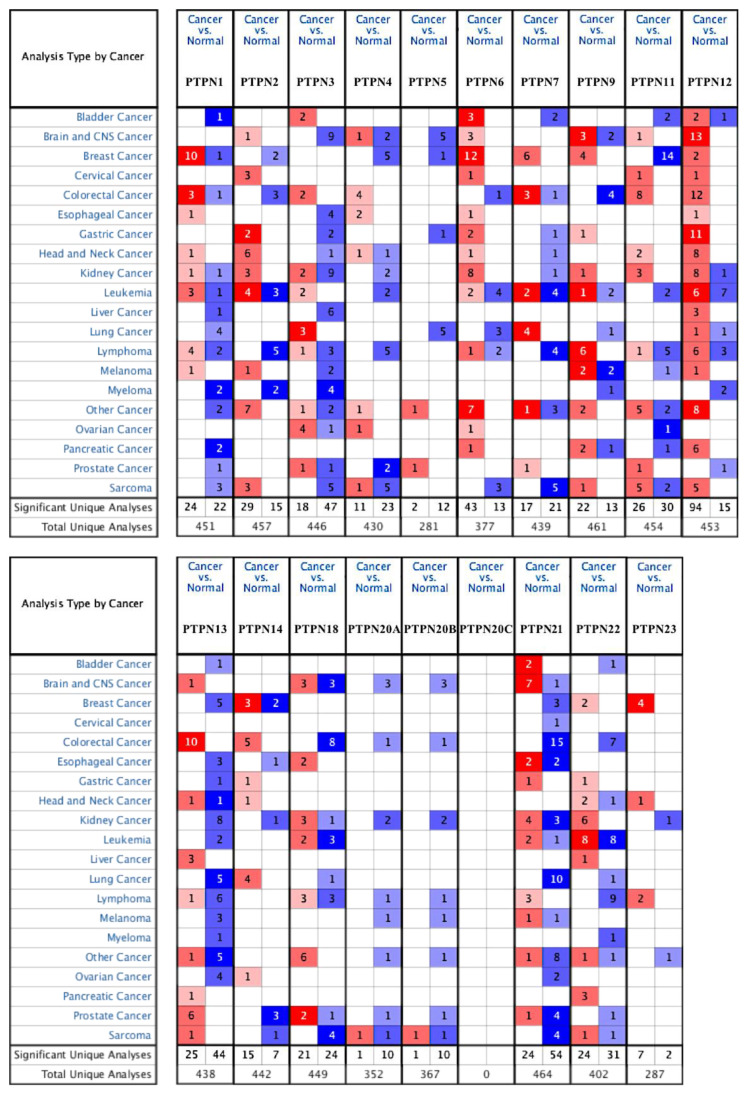
Transcription levels of protein tyrosine phosphatase non-receptor type (PTPN) family genes in different types of cancers. The thresholds of the *p* value and fold change were as follows: *p* = 0.01, fold change = 1.5, gene rank = 10%.

**Figure 2:**
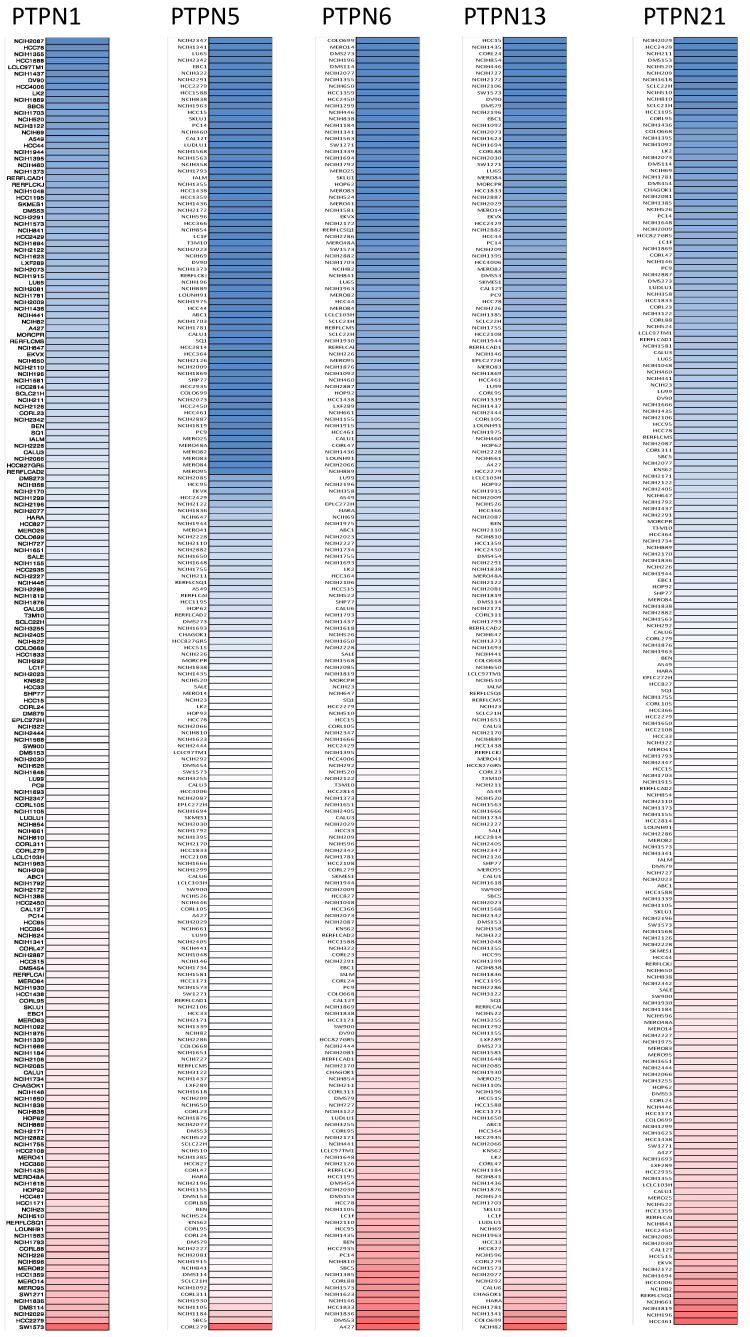
Gene expression levels of protein tyrosine phosphatase non-receptor type (*PTPN*) family members in lung cancer cell lines (from the CCLE database). The blue blocks indicate underexpression, whereas red blocks represent overexpression.

First, we used the ONCOMINE database to compare levels of different *PTPN* members in various cancers and normal tissue samples (Figure 1). Results showed that *PTPN3*, *PTPN7*, *PTPN12*, and *PTPN14* were expressed at higher levels, while expressions of *PTPN1*, *PTPN5*, *PTPN6*, *PTPN9*, *PTPN12*, *PTPN13*, *PTPN18*, *PTPN21*, and *PTPN22* were downregulated in lung cancer. The CCLE dataset was used to investigate mRNA expression levels in cancer cell lines in order to confirm the involvement of these PTPN family members. Values of the expression profiles were log-transformed and then displayed using a heatmap (Figure 2). Additionally, we further checked the analyses that met the threshold for PTPN genes according to the histopathological type of lung cancer (Supplementary Table S1). These datasets showed that *PTPN1/5/6/9/13/21* expressions were significantly lower in LUAD tissues with the following thresholds: *p* value = 0.01; fold change = 1.5; and gene rank = 10%. These results suggested that these PTPN genes may play roles as tumor-suppressor genes in LUAD patients.

### 3.2. Prognostic Values of PTPN Family Members in LUAD Patients

**Figure 3:**
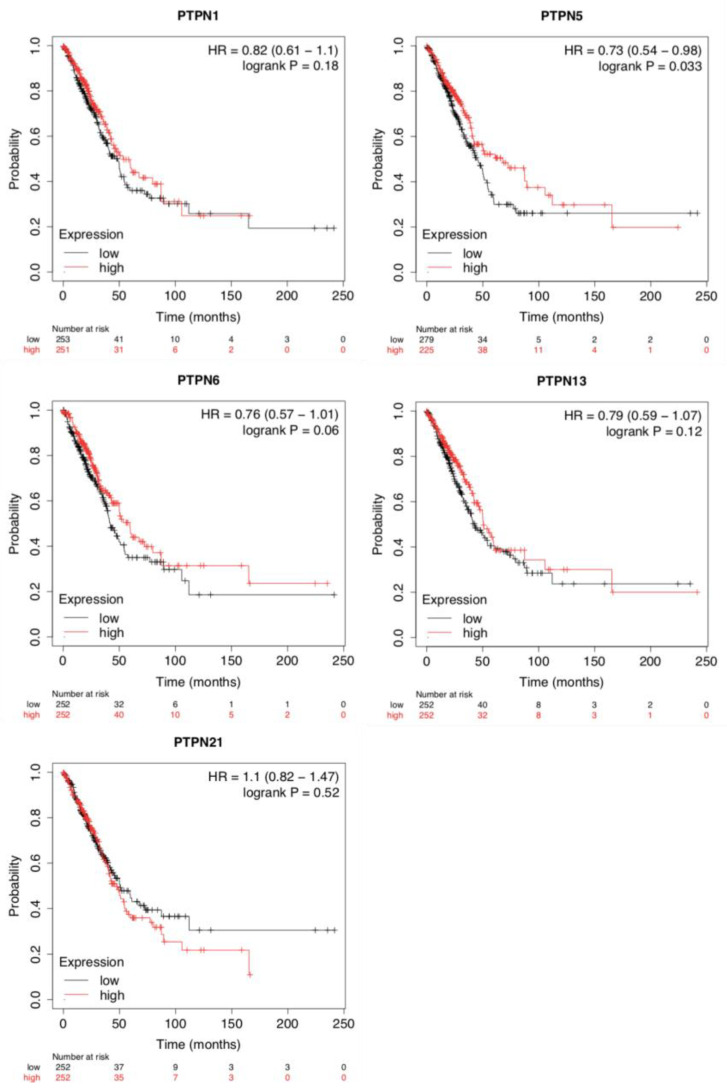
Prognostic values of different expressed protein tyrosine phosphatase non-receptor type *(PTPN*) family members in overall survival of lung adenocarcinoma (LUAD) patients (Kaplan–Meier plotter).

To evaluate the impacts of *PTPNs* at different expression levels on the progression of LUAD, we assessed correlations between these *PTPN* members and clinical outcomes using a KM plotter analysis (Figure 3). Survival curves revealed that LUAD patients with high transcription levels of *PTPN1*, *PTPN5*, *PTPN6*, *PTPN13*, and *PTPN21* were associated with longer OS. Results indicated that high *PTPN1/5/6/13/21* mRNA expression levels were related to a better prognosis of LUAD. Details of other PTPN genes are illustrated in Supplementary Table S2.

### 3.3. Relationships between PTPN Genes and Clinicopathological Parameters of LUAD Patients

**Figure 4:**
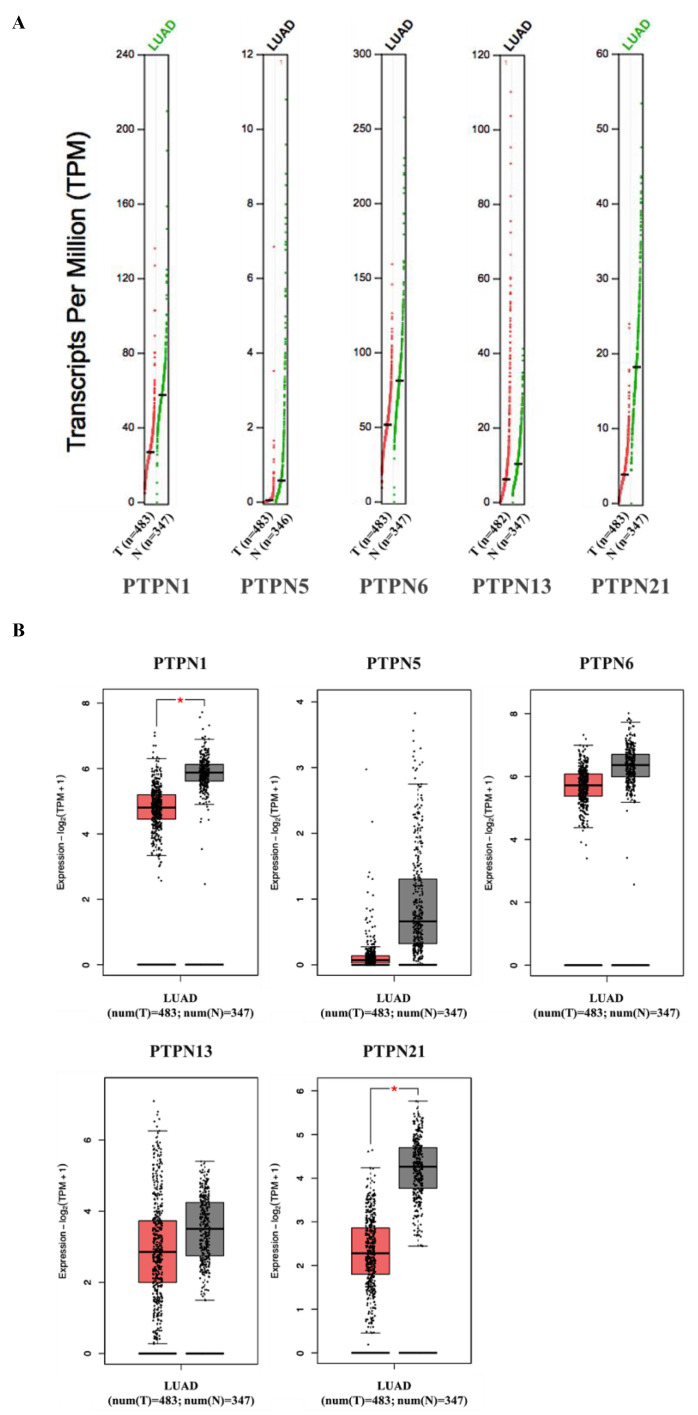
(**A**,**B**) Expressions of PTPN members in LUAD patients and normal tissue via the GEPIA 2 platform. The q-value cut-off was set to 0.01. The red star in the pictures indicates a significant difference between LUAD and normal tissues.

**Figure 5:**
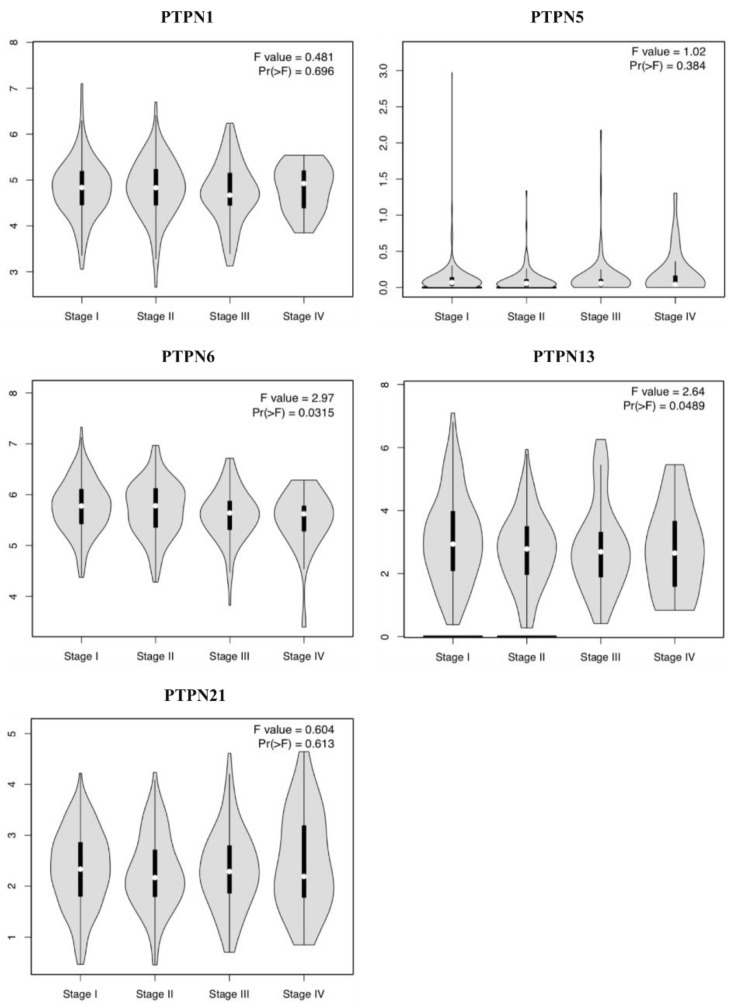
Correlations between expressions of protein tyrosine phosphatase non-receptor type (*PTPN*) family members and pathological stage in patients with lung adenocarcinoma (LUAD) via the GEPIA database.

Furthermore, we compared transcriptional levels of PTPNs between LUAD and normal samples by interrogating the GEPIA dataset (Figure 4). We found that transcription levels of *PTPN1* and *PTPN21* in LUAD tissues were significantly lower than those in normal tissues, while transcription levels of *PTPN5*, *PTPN6*, and *PTPN13* did not significantly differ between LUAD and normal tissues. Additionally, we also evaluated correlations between PTPN gene expression levels and the pathological stage of LUAD patients, and we discovered that expression levels of *PTPN6* and *PTPN13* were significantly correlated with the tumor stage of LUAD patients, while expression levels of *PTPN1*, *PTPN5*, and *PTPN21* were not (Figure 5). According to the above results, it was confirmed that *PTPN1*, *PTPN5*, *PTPN6*, *PTPN13*, and *PTPN21* were associated with the clinicopathological features of LUAD.

### 3.4. Genetic Alterations, Co-Expression, and Functional Enrichment Analyses of PTPNs in LUAD Patients

**Figure 6:**
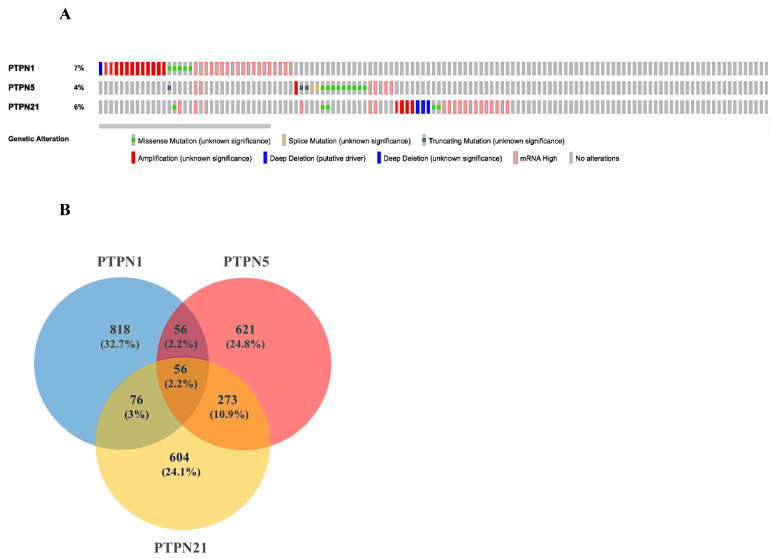

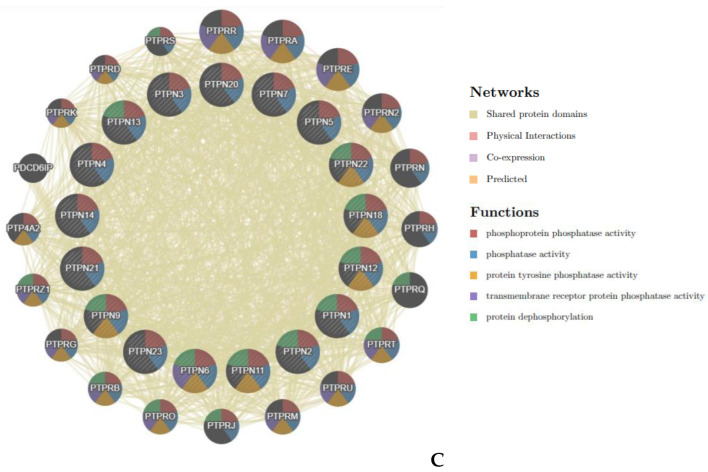
Genetic alterations, co-expression, and functional enrichment analysis of protein tyrosine phosphatase non-receptor type (*PTPN*) family members in lung adenocarcinoma (LUAD) patients. (**A**) Genetic alterations in *PTPN* family members in LUAD (from cBioPortal). (**B**,**C**) Common genes among *PTPN1*, *PTPN5*, and *PTPN21* (from Venny 2.0).

Based on the above results, we selected *PTPN1*, *PTPN5*, and *PTPN21* to further conduct a comprehensive analysis of the molecular characteristics. First, we used the cBioPortal online tool for TCGA LUAD cohort to analyze genetic alterations of these PTPN genes. Results showed that genetic alterations of PTPNs had different types, including missense mutations, splice mutations, truncating mutations, deep deletions, high mRNA expression, and amplifications. The genetic variation rate of *PTPN1* among 503 cases was 7%, which was the most frequently altered gene in these PTPN members, which included missense mutations, deep deletions, high mRNA expression, and amplifications. Others with high rates were *PTPN5* (4%) and *PTPN21* (6%) (Figure 6A).

Next, we input the genetic data of PTPNs from cBioPortal into Venny 2.0 and calculated the overlap rate of each group. Results revealed that there were 56 genes in common among *PTPN1*, *PTPN5*, and *PTPN21* (Figure 6B). Moreover, we analyzed the Gene Ontology (GO) in DAVID to identify enriched biological roles of PTPNs and predict their functions. The GO analysis was grouped into three aspects, including molecular functions (MFs), cellular components (CCs), and biological processes (BPs) (Supplementary Table S3C–F). We discovered that the BPs of *PTPN1*, *PTPN5*, and *PTPN21* were significantly highly enriched in positive regulation of GTPase activity, regulation of small GTPase-mediated signal transduction, response to mechanical stimuli, vasculogenesis, response to estrogen, organ morphogenesis, regulation of stress fiber assembly, mitogen-activated protein kinase (MAPK) cascade, cell migration involved in sprouting angiogenesis, and positive regulation of angiogenesis. These items may be related to the regulation of cell differentiation and cell migration of LUAD. In the CC group, the most significantly highly enriched items included cytoplasm, focal adhesion, intracellular, cytoskeleton, extracellular matrix, cell cortex, lamellipodium, transforming growth factor beta receptor homodimeric complex, and nuclear outer membrane. As for MFs, changes were mainly enriched in GTPase activator activity, heparin binding, protein binding, calcium ion binding, and lipid binding.

### 3.5. Gene-Gene Interaction (GGI) Networks of PTPNs in LUAD

Next, we used the GeneMANIA database to construct GGI networks of *PTPN1*, *PTPN5*, and *PTPN21*, and further analyzed their functions. As shown in Figure 6A–C, each group had 20 nodes, which, respectively represented genes that were strongly related to *PTPN1*, *PTPN5*, and *PTPN21* in terms of physical interactions, co-expression, predictions, co-localization, pathways, genetic interactions, and shared protein domains. The top five genes significantly associated with *PTPN1* were *NOX4* (NADPH oxidase 4), *CTH* (cystathionine gamma-lyase), *JAK2* (Janus kinase 2), *TRPV6* (transient receptor potential), and *FCGR2A* (Fc fragment of IgG receptor II-a). A further functional analysis showed that these genes were involved in the *JAK-STAT* pathway, and it was indicated that *PTPN1* was related to cell proliferation and cell differentiation (Figure 6A). In addition, Figure 6B shows that the main genes associated with *PTPN5* included *CAPN1* (calpain 1), *PTPRN* (protein tyrosine phosphatase receptor type N), *GRIN1* (glutamate ionotropic receptor NMDA type subunit 1), *GRIN2B* (glutamate ionotropic receptor NMDA type subunit 2B), and *MAPK3* (mitogen-activated protein kinase 3). From results of the functional analysis, we discovered that *PTPN5* was related to the transmission process of protein kinase signaling (Figure 6B). Moreover, Figure 6C also reveals that *KIF1C* (kinesin family member 1C), *SRC* (SRC proto-oncogene, non-receptor tyrosine kinase), *NRG3* (neuregulin 3), *BMX* (BMX non-receptor tyrosine kinase), and *PARD6B* (par-6 family cell polarity regulator beta) were genes highly associated with *PTPN21*. According to the functional analysis, we observed that *PTPN21* was primarily correlated with the activity of protein tyrosine kinase and cell migration (Figure 6C).

### 3.6. GGI Networks of PTPNs in LUAD

**Figure 7:**
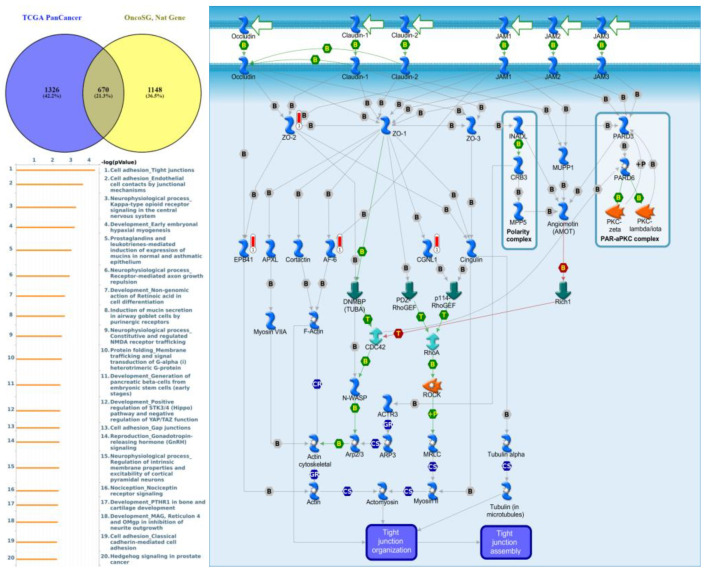
Expression of the protein tyrosine phosphatase non-receptor type 3 (PTPN3) signaling pathway in lung cancer (from Metacore). The functional analysis of “Cell adhesion_Tight junctions” was correlated with lung cancer development.

**Figure 8:**
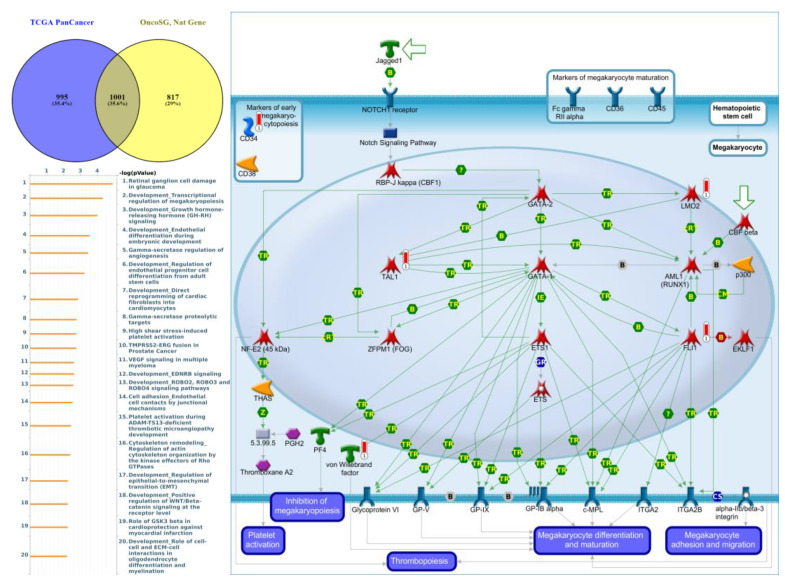
Expression of the protein tyrosine phosphatase non-receptor type 5 (PTPN5) signaling pathway in lung cancer (from Metacore). The functional analysis of “Development_Transcriptional regulation of megakaryopoiesis” was correlated with lung cancer development.

**Figure 9:**
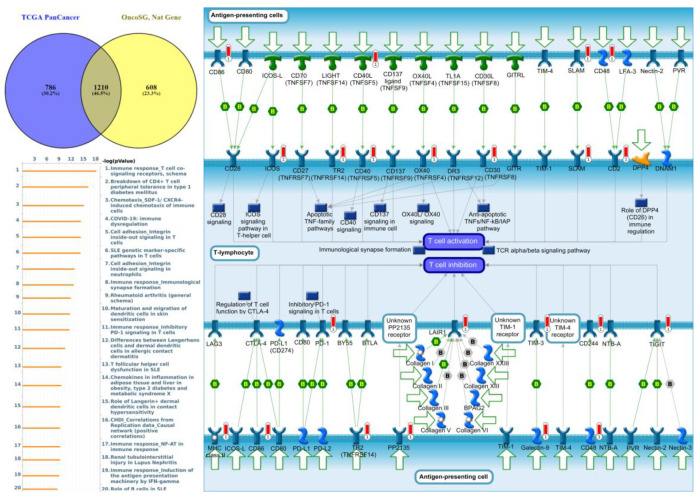
Expression of the protein tyrosine phosphatase non-receptor type 6 (PTPN6) signaling pathway in lung cancer (from Metacore). The functional analysis of “Immune response_T cell co-signaling receptors, schema” was correlated with lung cancer development.

**Figure 10:**
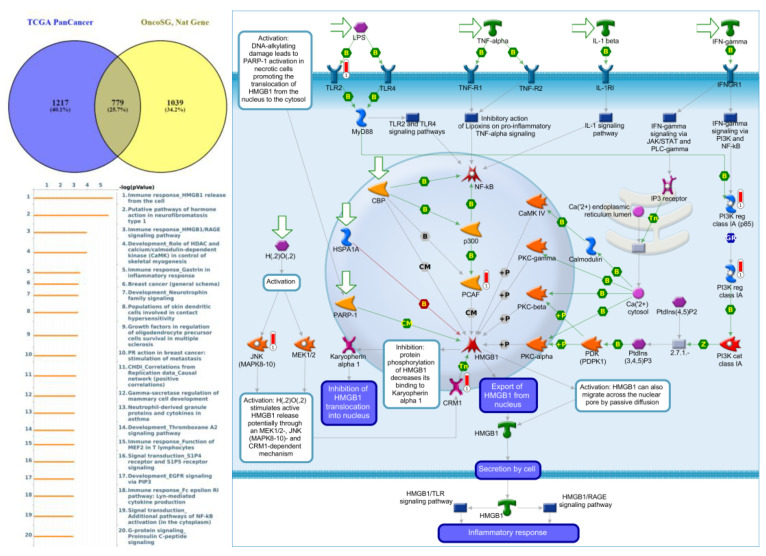
Expression of the protein tyrosine phosphatase non-receptor type 13 (PTPN13) signaling pathway in lung cancer (from Metacore). The functional analysis of “Immune response_HMGB1 release from the cell” was correlated with lung cancer development.

**Figure 11:**
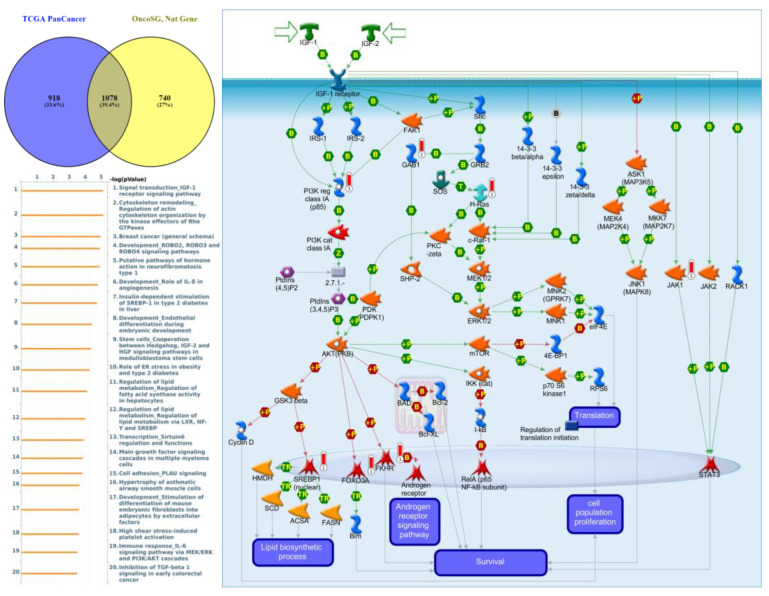
Expression of the protein tyrosine phosphatase non-receptor type 21 (PTPN21) signaling pathway in lung cancer (from Metacore). The functional analysis of “Signal transduction_IGF-1 receptor signaling pathway” was correlated with lung cancer development.

Genes co-expressed with *PTPN3* were correlated with “Cell adhesion_Tight junctions”, “Cell adhesion_Endothelial cell contacts by junctional mechanisms”, and “Neurophysiological process_Kappa-type opioid receptor signaling in the central nervous system” (Figure 7, Supplementary Table S4). Genes co-expressed with *PTPN5* were correlated with “Retinal ganglion cell damage in glaucoma”, “Development_Transcriptional regulation of megakaryopoiesis”, and “Development_Growth hormone-releasing hormone (GH-RH) signaling” (Figure 8, Supplementary Table S5). Genes co-expressed with *PTPN6* were correlated with “Immune response_T cell co-signaling receptors, schema”, “Breakdown of CD4^+^ T cell peripheral tolerance in type 1 diabetes mellitus”, and “Chemotaxis_SDF-1/CXCR4-induced chemotaxis of immune cells” (Figure 9, Supplementary Table S6). Genes co-expressed with *PTPN13* were correlated with “Immune response_HMGB1 release from the cell”, “Putative pathways of hormone action in neurofibromatosis type 1”, and “Immune response_HMGB1/RAGE signaling pathway” (Figure 10, Supplementary Table S7). Genes co-expressed with PTPN21 were correlated with “Signal transduction_IGF-1 receptor signaling pathway”, “Cytoskeleton remodeling_Regulation of actin cytoskeleton organization by the kinase effectors of Rho GTPases”, and “Breast cancer (general schema)” (Figure 11, Supplementary Table S8). Meanwhile, we also use Human Protein Atlas database to detect the protein expression of PTPN family members (Supplementary Figure S1), and validate above data via cBioPortal (Supplementary Figure S2) and GSEA as well as KEGG database (Supplementary Figure S3).

## 4. Discussion

Lung cancer is composed of SCLC and NSCLC. NSCLC comprises around 85% of lung cancer cases, and it is further classified into three subtypes, squamous-cell carcinoma, adenocarcinoma, and large-cell carcinoma. LUAD is the main subtype of NSCLC, accounting for 40% of all cases of lung cancer. Therefore, it is especially important to explore effective biomarkers and exploit novel therapeutic developments [41,42,43,44,45]. By applying advances in high-throughput screening for cancer transcriptome profiling [46,47,48,49], alterations of transcriptome patterns of encoded PTPN gene families have been discovered to be significantly associated with several types of malignancies. However, since previous research has yet to elaborate on the roles of PTPN family genes in LUAD, this study may serve as the first and foremost work to specifically examine roles of PTPN individuals in this disease, prior to giving a more extensive and incisive understanding about potential therapeutic and prognostic values in view of the benefits they offer to LUAD patients.

A previous study demonstrated that PPDPF induces hyperactive STAT3 by interfering with STAT3-PTPN1 interactions in LUAD [50]. The diversity of PTPN2’s effects in different types of tumor makes it a potential target for tumor immunotherapy [51]. PTPN3 is a potential target for new cancer immunotherapy that has a dual effect of T cell activation [52]. MiR-375 directly interfered with the expression of PTPN4, which in turn stabilized phosphorylated STAT3 in castration-resistant prostate cancer [53]. Increased expression of PTPN5 was seen in canine oral tumor groups [54]. STAT3 Lys140 hypermethylation caused by a Jmjd1c deletion inhibited interaction with the Ptpn6 phosphatase [55]. PTPN7 is amplified in myeloid malignancies and deleted in lymphoproliferative disorders [56]. miR-126-3p mediated the inhibitory effect of exosomes on A549 cells by negative regulation of PTPN9 in NSCLC cells [57]. Concomitant deletion of Ptpn6 and Ptpn11 in T cells failed to improve anticancer responses [58]. The miR-940/PTPN12 axis could be a potential drug target to treat esophageal squamous cell carcinoma [59]. miR-200b, ZEB2, and PTPN13 are downregulated in colorectal carcinoma with serosal invasion [60]. PTPN14 loss-of-function variants were associated with high risks of cervical cancer and an early age at diagnosis [61]. Nuclear import of PTPN18 inhibited breast cancer metastasis mediated by MVP and importin β2 [62]. PTPN20 was associated with a worse OS of digestive tract cancers [63]. PTPN21 was upregulated in glioma tissues, and a high PTPN21 level predicted poor survival rates in glioma patients [64]. PTPN22 negatively modulates platelet function and thrombus formation [65]. PTPN23 was recently associated with several human epithelial cancers [66].

Our bioinformatics analysis indicated that PTPN gene expressions were found to be involve in tumor multi-stage progression. According to various database analyses, *PTPN3*, *PTPN7*, *PTPN12*, and *PTPN14* were expressed at higher levels, while expressions of *PTPN1*, *PTPN5*, *PTPN6*, *PTPN9*, *PTPN12*, *PTPN13*, *PTPN18*, *PTPN21*, and *PTPN22* were downregulated in lung cancer. Survival curves revealed that LUAD patients with high transcription levels of *PTPN1*, *PTPN5*, *PTPN6*, *PTPN13*, and *PTPN21* were associated with longer OS. These data suggest that *PTPN1*, *PTPN5*, *PTPN6*, *PTPN13*, and *PTPN21* are associated with clinicopathological features of LUAD.

Meanwhile, GO indicated that genes co-expressed with *PTPN1*, *PTPN5*, and *PTPN21* were significantly highly enriched in the positive regulation of GTPase activity, regulation of small GTPase-mediated signal transduction, response to mechanical stimuli, vasculogenesis, response to estrogen, organ morphogenesis, regulation of stress fiber assembly, MAPK cascade, cell migration involved in sprouting angiogenesis, and positive regulation of angiogenesis. The MetaCore analysis of lung cancer patients also suggested that *PTPN3* signaling was correlated with “Cell adhesion_Tight junctions”, *PTPN5* signaling was correlated with “Development_Transcriptional regulation of megakaryopoiesis”, *PTPN6* signaling was correlated with “Development_Transcriptional regulation of megakaryopoiesis”, *PTPN13* signaling was correlated with “Immune response_HMGB1 release from the cell”, and *PTPN21* signaling was correlated with “Signal transduction_IGF-1 receptor signaling pathway”.

These results were consistent with previous findings. However, there are a few limitations of our research. The data we analyzed in this study were derived from high throughput databases. Therefore, further cellular studies are required to verify our findings and investigate possible mechanisms, molecular interactions, and clinical applications of various PTPN family genes involved in cancer development.

## 5. Conclusions

As previous research was yet to elaborate the roles of PTPN family genes in LUAD, this study may serve as the first and foremost work that specifically examined roles of PTPN individuals in this disease, prior to giving a more extensive and incisive understanding of potential therapeutic and prognostic values in view of the benefits they offer to LUAD patients.

## Data Availability

The datasets used and analyzed during the current study are available from the corresponding author upon reasonable request.

## Supplementary Materials

The following supporting information can be downloaded at: https://www.mdpi.com/article/10.3390/jpm12121947/s1. Figure S1. Protein expression levels of members of the protein tyrosine phosphatase non-receptor type (PTPN) family in lung cancer specimens from the Human Protein Atlas; Figure S2. Correlations between different protein tyrosine phosphatase non-receptor type (PTPN) family members in lung cancer (from cBioPortal); insignificant correlations are marked by crosses; Figure S3. Hallmark and KEGG signaling pathway analysis of protein tyrosine phosphatase non-receptor type (PTPN) family members in lung cancer via the GSEA databse; Table S1 Significant changes in expressions of protein tyrosine phosphatase non-receptor type (PTPN) family members at the transcription level between different types of lung cancer (from the ONCOMINE database); Table S2 Prognostic roles of protein tyrosine phosphatase non-receptor type (PTPN) family members in lung adenocarcinoma (LUAD); Table S3A-D. (C) GO analysis of the protein tyrosine phosphatase non-receptor type (PTPN) family, (D) PTPN1, (E) PTPN5, (F) PTPN21. The top 20 enriched classifications for biological processes, cellular components, and molecular functions are shown (from DAVID); Table S4: Pathway analysis of genes coexpressed with PTPN3 (protein tyrosine phosphatase non-receptor type 3) (from public lung cancer databases using the MetaCore database (with *p* < 0.05 set as the cutoff value); Table S5: Pathway analysis of genes coexpressed with PTPN5 (protein tyrosine phosphatase non-receptor type 5) from public lung cancer databases using the MetaCore database (with *p* < 0.05 set as the cutoff value); Table S6: Pathway analysis of genes coexpressed with PTPN6 (protein tyrosine phosphatase non-receptor type 6) from public lung cancer databases using the MetaCore database (with *p* < 0.05 set as the cutoff value); Table S7: Pathway analysis of genes coexpressed with PTPN13 (protein tyrosine phosphatase non-receptor type 13) from public lung cancer databases using the MetaCore database (with *p* < 0.05 set as the cutoff value); Table S8: Pathway analysis of genes coexpressed with PTPN21 (protein tyrosine phosphatase non-receptor type 21) from public lung cancer databases using the MetaCore database (with *p* < 0.05 set as the cutoff value).
