# Supplementary material for: Novel Potential Therapeutic Targets of PTPN Families for Lung Cancer
Source: J Pers Med. 2022 Nov 23;12(12):1947. doi: 10.3390/jpm12121947 (PMC9784538; doi:10.3390/jpm12121947)
Supplement: Supplementary file 1 [file jpm-12-01947-s001.zip › jpm-2018584-supplementary.pdf]

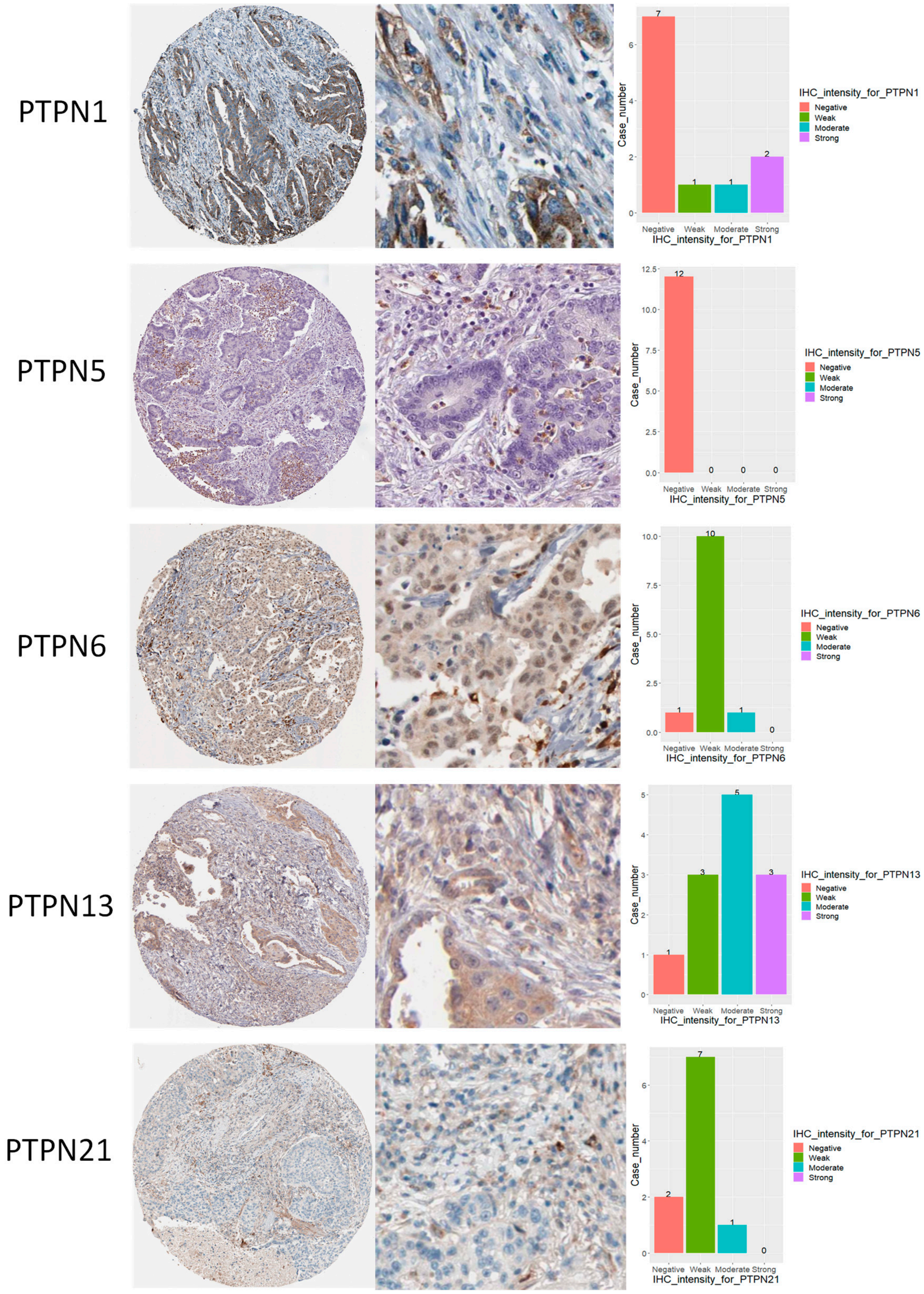

Figure S1. Protein expression levels of members of the protein tyrosine phosphatase non-receptor type (PTPN) family in lung cancer specimens from the Human Protein Atlas.

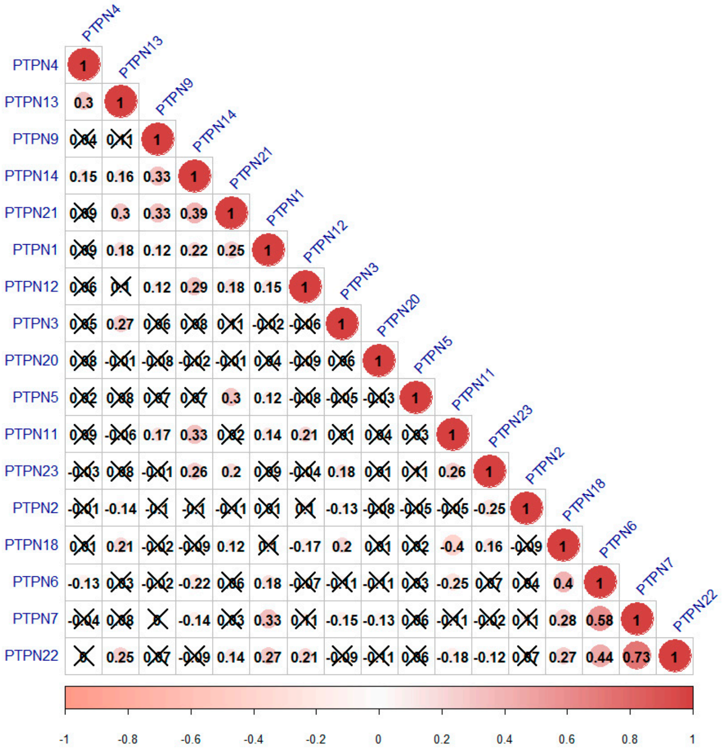

Figure S2. Correlations between different protein tyrosine phosphatase non-receptor type (*PTPN*) family members in lung cancer (from cBioPortal); insignificant correlations are marked by crosses.

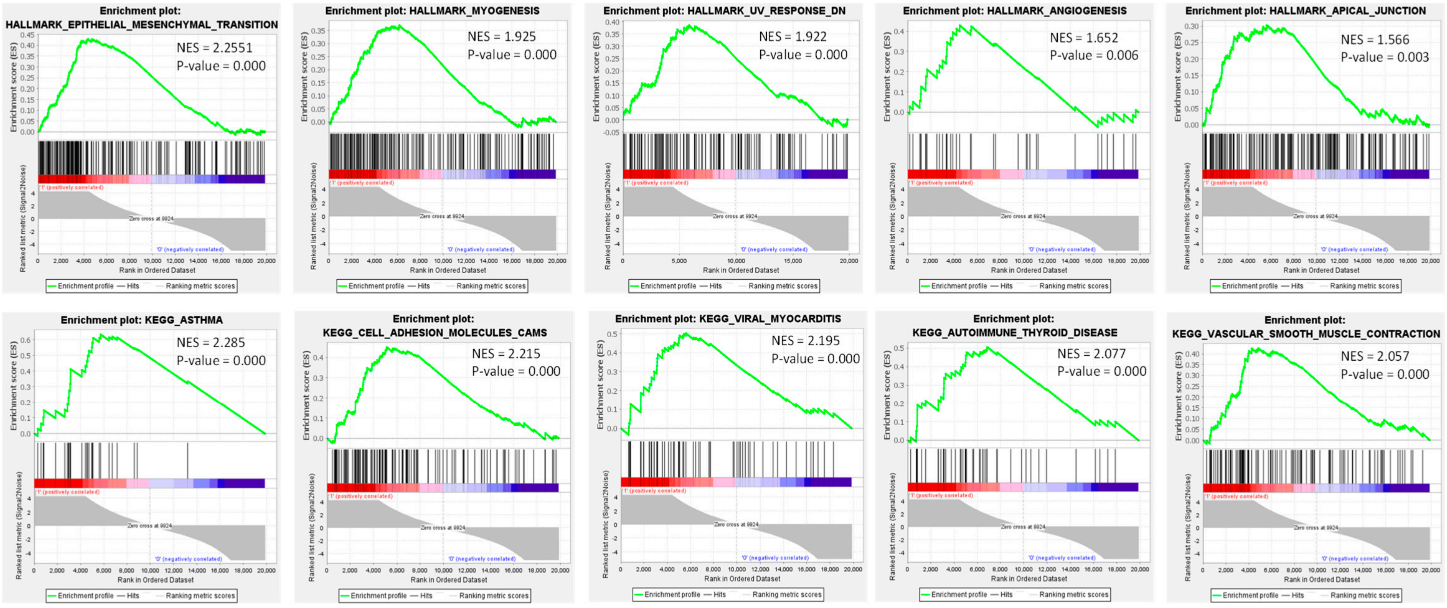

Figure S3. Hallmark and KEGG signaling pathway analysis of protein tyrosine phosphatase non-receptor type (*PTPN*) family members in lung cancer via the GSEA databse.

Table S1 Significant changes in expressions of protein tyrosine phosphatase non-receptor type (*PTPN*) family members at the transcription level between different types of lung cancer (from the ONCOMINE database)

	Types of LUAD vs. normal	Fold change	t-test	*P*-value	Ref
**PTPN1**	Lung Adenocarcinoma	-3.285	-3.97	4.51E-04	
	Lung Adenocarcinoma	-1.581	-7.37	4.88E-11	
	Squamous Cell Lung Carcinoma	-1.632	-8.016	3.71E-11	
	Large Cell Lung Carcinoma	-1.8	-6.647	2.45E-07	
**PTPN2**	NA	NA	NA	NA	NA
**PTPN3**	NA	NA	NA	NA	NA
**PTPN4**	NA	NA	NA	NA	NA
**PTPN5**	Lung Adenocarcinoma	-4.26	-6.04	1.70E-07	
	Lung Adenocarcinoma	-1.803	-9.322	1.22E-14	
	Squamous Cell Lung Carcinoma	-1.767	-8.524	2.20E-13	
	Large Cell Lung Carcinoma	-1.649	-6.422	7.83E-09	
	Lung Adenocarcinoma	-6.004	-10.285	1.37E-10	
**PTPN6**	Squamous Cell Lung Carcinoma	-1.869	-5.324	3.55E-04	
	Lung Adenocarcinoma	-1.622	-7.343	1.30E-10	
	Large Cell Lung Carcinoma	-2.411	-7.653	7.78E-08	
**PTPN7**	NA	NA	NA	NA	NA
**PTPN9**	Lung Adenocarcinoma	-1.722	-4.927	1.89E-06	
**PTPN11**	NA	NA	NA	NA	NA
**PTPN12**	Lung Carcinoid Tumor	-9.125	-7.994	6.54E-08	
**PTPN13**	Small Cell Lung Carcinoma	-11.733	-6.717	1.08E-06	
	Lung Carcinoid Tumor	-22.837	-8.772	3.86E-10	
	Lung Adenocarcinoma	-3.745	-4.309	1.61E-04	
	Lung Adenocarcinoma	-2.507	-7.94	2.74E-11	
	Large Cell Lung Carcinoma	-5.627	-8.672	1.55E-08	
**PTPN14**	NA	NA	NA	NA	NA
**PTPN18**	Small Cell Lung Carcinoma	-3.869	-4.526	9.25E-05	
**PTPN20A**	NA	NA	NA	NA	NA
**PTPN20B**	NA	NA	NA	NA	NA
**PTPN20C**	NA	NA	NA	NA	NA
**PTPN21**	Lung Adenocarcinoma	-4.285	-11.318	6.24E-19	
	Squamous Cell Lung Carcinoma	-6.82	-14.166	2.36E-19	
	Large Cell Lung Carcinoma	-6.765	-11.979	5.29E-13	
	Lung Adenocarcinoma	-3.169	-7.027	1.85E-07	
	Large Cell Lung Carcinoma	-3.035	-3.901	3.00E-03	
	Small Cell Lung Carcinoma	-4.051	-3.645	4.00E-03	
	Squamous Cell Lung Carcinoma	-3.683	-4.353	1.00E-03	
	Lung Adenocarcinoma	-3.434	-4.589	3.00E-03	
	Lung Adenocarcinoma	-3.08	-13.729	1.05E-13	
	Lung Adenocarcinoma	-2.534	-4.702	9.03E-06	
**PTPN22**	Large Cell Lung Carcinoma	-2.631	-7.478	1.07E-07	
**PTPN23**	NA	NA	NA	NA	NA

	Types of LUAD vs. normal	Fold change	t-test	*P*-value	Ref
**PTPN1**	NA	NA	NA	NA	NA
**PTPN2**	NA	NA	NA	NA	NA
**PTPN3**	Lung Adenocarcinoma	1.772	5.563	2.41E-06	
	Lung Adenocarcinoma	1.650	4.867	4.23E-05	
	Lung Adenocarcinoma	1.635	4.706	1.48E-05	
**PTPN4**	NA	NA	NA	NA	NA
**PTPN5**	NA	NA	NA	NA	NA
**PTPN6**	NA	NA	NA	NA	NA
**PTPN7**	Large Cell Lung Carcinoma	4.067	10.665	2.72E-06	
	Lung Adenocarcinoma	2.372	5.981	5.49E-06	
	Squamous Cell Lung carcinoma	1.625	3.080	0.004	
	Squamous Cell Lung carcinoma	6.748	4.298	6.76E-05	
**PTPN9**	NA	NA	NA	NA	NA
**PTPN11**	NA	NA	NA	NA	NA
**PTPN12**	Squamous Cell Lung carcinoma	1.980	7.852	1.15E-10	
**PTPN13**	NA	NA	NA	NA	NA
**PTPN14**	Lung Adenocarcinoma	1.585	4.746	5.90E-05	
	Squamous Cell Lung carcinoma	2.331	4.125	5.74E-04	
	Large Cell Lung Carcinoma	2.833	4.139	1.00E-02	
	Squamous Cell Lung carcinoma	2.906	3.689	4.82E-04	
**PTPN18**	NA	NA	NA	NA	NA
**PTPN20A**	NA	NA	NA	NA	NA
**PTPN20B**	NA	NA	NA	NA	NA
**PTPN20C**	NA	NA	NA	NA	NA
**PTPN21**	NA	NA	NA	NA	NA
**PTPN22**	NA	NA	NA	NA	NA
**PTPN23**	NA	NA	NA	NA	NA

Table S2 Prognostic roles of protein tyrosine phosphatase non-receptor type (*PTPN*) family members in lung adenocarcinoma (LUAD)

Gene	Univariate analysis	
	HR (95% CI)	P
**PTPN1**	0.81 (0.6-1.08)	0.15
**PTPN2**	0.81 (0.57-1.15)	0.24
**PTPN3**	0.77 (0.54-1.1)	0.16
**PTPN4**	0.58 (0.41-0.82)	0.0017
**PTPN5**	0.6 (0.44-0.81)	0.00086
**PTPN6**	0.56 (0.4-0.8)	0.0011
**PTPN7**	0.68 (0.5-0.92)	0.012
**PTPN9**	1.44 (1.05-1.97)	0.023
**PTPN11**	1.5 (1.11-2.03)	0.0082
**PTPN12**	1.44 (1.04-1.97)	0.025
**PTPN13**	0.7 (0.51-0.97)	0.032
**PTPN14**	1.27 (0.95-1.7)	0.1
**PTPN18**	0.69 (0.5-0.95)	0.02
**PTPN20**	0.72 (0.54-0.98)	0.035
**PTPN21**	1.22 (0.86-1.72)	0.26
**PTPN22**	0.7 (0.52-0.94)	0.017
**PTPN23**	0.71 (0.51-0.97)	0.033

Table S3A-D. (A) GO analysis of the protein tyrosine phosphatase non-receptor type (*PTPN*) family, (B) PTPN1, (C) PTPN5, (D) PTPN21. The top 20 enriched classifications for biological processes, cellular components, and molecular functions are shown (from DAVID).

A

Biological Process

Term	Count	%	P-Value
Positive regulation of GTPase activity	9	16.7	2.40E-04
Regulation of small GTPase mediated signal transduction	5	9.3	6.70E-04
Response to mechanical stimulus	4	7.4	7.20E-04
Vasculogenesis	3	5.6	1.20E-02
Response to estrogen	3	5.6	1.60E-02
Organ morphogenesis	3	5.6	3.10E-02
Regulation of stress fiber assembly	2	3.7	3.80E-02
MAPK cascade	4	7.4	4.30E-02
Cell migration involved in sprouting angiogenesis	2	3.7	4.40E-02
Positive regulation of angiogenesis	3	5.6	4.60E-02
Embryonic hemopoiesis	2	3.7	4.70E-02
Cell differentiation	5	9.3	4.80E-02
Nervous system development	4	7.4	5.40E-02
Actin cytoskeleton organization	3	5.6	5.70E-02
Negative regulation of peptidyl-serine phosphorylation	2	3.7	6.60E-02
Negative regulation of canonical Wnt signaling pathway	3	5.6	8.50E-02
Regulation of angiogenesis	2	3.7	8.80E-02
Response to hypoxia	3	5.6	9.30E-02
Positive regulation of epithelial to mesenchymal transition	2	3.7	9.40E-02

Cellular Component

Term	Count	%	P-Value
Focal adhesion	9	16.7	1.40E-05
Transforming growth factor beta receptor homodimeric complex	2	3.7	1.10E-02
Cytoskeleton	5	9.3	2.10E-02
Cytoplasm	22	40.7	4.60E-02
Cell cortex	3	5.6	4.80E-02
Extracellular matrix	4	7.4	5.20E-02
Nuclear outer membrane	2	3.7	6.60E-02
Lamellipodium	3	5.6	7.60E-02
Intracellular	8	14.8	8.30E-02

Molecular Function

Term	Count	%	P-Value
GTPase activator activity	6	11.1	1.30E-03
Heparin binding	4	7.4	1.10E-02
Protein binding	34	63	2.20E-02
Calcium ion binding	6	11.1	5.60E-02
Lipid binding	3	5.6	7.10E-02

B

Biological Process

Term	Count	%	P-Value
Immune response	86	8.8	1.00E-28
Inflammatory response	77	7.9	1.40E-25
Adaptive immune response	48	4.9	3.40E-25
Leukocyte migration	38	3.9	2.40E-19
Innate immune response	70	7.2	8.90E-18
Signal transduction	129	13.2	1.70E-17
Positive regulation of GTPase activity	80	8.2	1.20E-16
T cell costimulation	28	2.9	4.10E-16
Regulation of immune response	39	4	3.20E-14
Chemotaxis	29	3	1.20E-11
Positive regulation of T cell proliferation	20	2	6.20E-11
Cell surface receptor signaling pathway	43	4.4	1.40E-10
Transmembrane receptor protein tyrosine kinase signaling pathway	24	2.5	3.40E-10
Cell adhesion	58	5.9	3.90E-10
T cell activation	17	1.7	6.00E-10
Positive regulation of interferon-gamma production	16	1.6	4.10E-09
T cell receptor signaling pathway	28	2.9	6.50E-09
Cellular defense response	18	1.8	7.50E-09
Cellular response to lipopolysaccharide	24	2.5	1.00E-08
Interferon-gamma-mediated signaling pathway	19	1.9	1.10E-08

Cellular Component

Term	Count	%	P-Value
Plasma membrane	344	35.1	9.60E-25
External side of plasma membrane	54	5.5	1.20E-22
Integral component of plasma membrane	155	15.8	2.30E-20
Integral component of membrane	361	36.9	9.50E-13
Membrane	185	18.9	2.40E-12
Cell surface	67	6.8	3.80E-11
T cell receptor complex	9	0.9	1.20E-06
Extrinsic component of cytoplasmic side of plasma membrane	16	1.6	1.20E-06
Immunological synapse	11	1.1	4.70E-06
Membrane raft	28	2.9	6.20E-06
Focal adhesion	42	4.3	9.50E-06
Actin filament	13	1.3	9.30E-05
Endocytic vesicle membrane	13	1.3	1.10E-04
Mitochondrial respiratory chain complex I	11	1.1	1.50E-04
Cytoskeleton	37	3.8	1.60E-04
Cell-cell junction	22	2.2	1.80E-04
Lamellipodium	21	2.1	1.80E-04
Mitochondrial inner membrane	41	4.2	3.00E-04
Ruffle membrane	13	1.3	8.60E-04
Cytosol	205	20.9	1.50E-03

Molecular Function

Term	Count	%	P-Value
Receptor activity	48	4.9	9.50E-18
Transmembrane signaling receptor activity	34	3.5	1.00E-08
Protein binding	518	52.9	1.80E-07
GTPase activator activity	37	3.8	2.40E-07
Guanyl-nucleotide exchange factor activity	21	2.1	1.80E-06
Phosphatidylinositol-4,5-bisphosphate 3-kinase activity	15	1.5	1.90E-06
Chemokine receptor activity	8	0.8	1.00E-05
Carbohydrate binding	26	2.7	2.00E-05
MHC class I protein binding	8	0.8	2.40E-05
SH3 domain binding	19	1.9	2.90E-05
Cytokine receptor activity	10	1	5.70E-05
Actin binding	31	3.2	7.80E-05
Non-membrane spanning protein tyrosine kinase activity	11	1.1	8.10E-05
Coreceptor activity	9	0.9	9.00E-05
IgG binding	6	0.6	1.20E-04
Protein tyrosine kinase activity	19	1.9	1.30E-04
C-C chemokine receptor activity	6	0.6	1.90E-04
Receptor binding	35	3.6	2.50E-04
G-protein coupled purinergic nucleotide receptor activity	6	0.6	4.50E-04
NADH dehydrogenase (ubiquinone) activity	10	1	4.50E-04

C

Biological Process

Term	Count	%	P-Value
Angiogenesis	39	4	3.50E-11
Extracellular matrix organization	34	3.5	9.50E-10
Vasculogenesis	17	1.7	9.50E-09
Cell adhesion	54	5.5	1.50E-08
Positive regulation of cardiac muscle cell proliferation	11	1.1	3.80E-08
Positive regulation of GTPase activity	59	6	2.00E-07
Outflow tract morphogenesis	13	1.3	2.00E-06
Calcium ion transport	16	1.6	4.90E-06
Heart looping	14	1.4	8.60E-06
Homophilic cell adhesion via plasma membrane adhesion molecules	23	2.4	1.50E-05
Heart development	25	2.6	1.70E-05
Sprouting angiogenesis	9	0.9	2.00E-05
Smooth muscle cell differentiation	7	0.7	2.00E-05
Positive regulation of phosphatidylinositol 3-kinase signaling	13	1.3	8.50E-05
Palate development	14	1.4	1.00E-04
Negative regulation of cell proliferation	39	4	1.10E-04
Positive regulation of cytosolic calcium ion concentration	19	1.9	1.40E-04
Positive regulation of gene expression	29	3	1.50E-04
Axon guidance	21	2.1	1.50E-04
In utero embryonic development	23	2.4	1.90E-04

Cellular Component

Term	Count	%	P-Value
Proteinaceous extracellular matrix	49	5	8.30E-15
Plasma membrane	296	30.3	1.60E-12
Integral component of plasma membrane	120	12.3	8.10E-09
Extracellular matrix	41	4.2	9.20E-09
Extracellular region	125	12.8	5.40E-07
Z disc	21	2.1	1.40E-06
Sarcolemma	17	1.7	3.90E-06
Extracellular space	102	10.4	2.00E-05
Cell surface	50	5.1	4.30E-05
Membrane raft	25	2.6	9.80E-05
Dendritic spine	16	1.6	1.20E-04
Integral component of membrane	307	31.4	1.70E-04
Focal adhesion	37	3.8	3.00E-04
Cell junction	41	4.2	4.40E-04
Elastic fiber	4	0.4	4.70E-04
Neuron spine	4	0.4	4.70E-04
Receptor complex	17	1.7	5.80E-04
Collagen trimer	14	1.4	6.20E-04
Cell-cell junction	20	2	9.60E-04
External side of plasma membrane	23	2.4	9.90E-04

Molecular Function

Term	Count	%	P-Value
Calcium ion binding	81	8.3	4.80E-12
Heparin binding	23	2.4	1.30E-05
Ras guanyl-nucleotide exchange factor activity	18	1.8	4.70E-05
Transcription factor activity, RNA polymerase II distal enhancer sequence-specific binding	13	1.3	8.00E-05
Transforming growth factor beta-activated receptor activity	5	0.5	1.80E-04
Metallopeptidase activity	13	1.3	5.80E-04
Integrin binding	15	1.5	6.30E-04
Transforming growth factor beta binding	6	0.6	8.00E-04
Ion channel binding	15	1.5	1.30E-03
Actin binding	27	2.8	1.30E-03
Transcriptional activator activity, RNA polymerase II core promoter proximal region sequence-specific binding	24	2.5	1.50E-03
Growth factor binding	7	0.7	1.70E-03
Scavenger receptor activity	9	0.9	2.20E-03
Transmembrane receptor protein tyrosine kinase activity	8	0.8	2.30E-03
Voltage-gated sodium channel activity	6	0.6	2.40E-03
GTPase activator activity	26	2.7	2.90E-03
ATPase activity, coupled to transmembrane movement of substances	8	0.8	5.40E-03
Receptor activity	21	2.1	5.40E-03
Muscle alpha-actinin binding	4	0.4	8.00E-03
Sulfotransferase activity	7	0.7	8.80E-03

D

Biological Process

Term	Count	%	P-Value
Mitochondrial translational elongation	28	2.9	1.10E-14
Mitochondrial translational termination	28	2.9	1.60E-14
Cell division	47	4.8	8.80E-09
Anaphase-promoting complex-dependent catabolic process	20	2	1.80E-08
Mitotic nuclear division	34	3.5	8.30E-07
Translation	34	3.5	1.30E-06
Mitochondrial electron transport, cytochrome c to oxygen	9	0.9	4.00E-06
Positive regulation of ubiquitin-protein ligase activity involved in regulation of mitotic cell cycle transition	16	1.6	8.00E-06
Mitotic cytokinesis	10	1	1.10E-05
Negative regulation of ubiquitin-protein ligase activity involved in mitotic cell cycle	15	1.5	1.60E-05
Mitochondrial respiratory chain complex I assembly	14	1.4	1.90E-05
Mitochondrial electron transport, NADH to ubiquinone	12	1.2	3.70E-05
Cell proliferation	39	4	4.80E-05
Hydrogen ion transmembrane transport	13	1.3	6.60E-05
Mitochondrial electron transport, ubiquinol to cytochrome c	7	0.7	6.90E-05
G1/S transition of mitotic cell cycle	17	1.7	7.90E-05
mRNA splicing, via spliceosome	27	2.8	1.10E-04
Regulation of ubiquitin-protein ligase activity involved in mitotic cell cycle	8	0.8	1.30E-04
Cell adhesion	44	4.5	1.70E-04
Nuclear import	7	0.7	2.20E-04

Cellular Component

Term	Count	%	P-Value
Mitochondrial inner membrane	78	8	6.60E-22
Mitochondrion	128	13	2.40E-12
Mitochondrial large ribosomal subunit	19	1.9	7.70E-12
Focal adhesion	53	5.4	2.10E-10
Mitochondrial intermembrane space	21	2.1	4.80E-10
Nucleoplasm	211	21.5	8.40E-10
Cytosol	240	24.5	2.70E-09
Nucleolus	79	8.1	4.20E-07
Ribosome	26	2.7	1.10E-06
Cytoplasm	327	33.3	6.50E-06
Methylosome	7	0.7	1.20E-05
Extracellular matrix	34	3.5	2.10E-05
Mitochondrial respiratory chain complex I	12	1.2	2.80E-05
U12-type spliceosomal complex	9	0.9	3.00E-05
Proteinaceous extracellular matrix	31	3.2	4.50E-05
Actin cytoskeleton	27	2.8	4.90E-05
Mitochondrial ribosome	9	0.9	5.50E-05
Midbody	19	1.9	9.50E-05
U4 snRNP	6	0.6	1.20E-04
SMN-Sm protein complex	7	0.7	1.30E-04

Molecular Function

Term	Count	%	P-Value
Protein binding	584	59.5	1.50E-19
Structural constituent of ribosome	36	3.7	3.80E-09
Poly(A) RNA binding	105	10.7	5.30E-09
Cytochrome-c oxidase activity	10	1	1.40E-05
Actin binding	31	3.2	1.20E-04
NADH dehydrogenase (ubiquinone) activity	11	1.1	1.50E-04
Heparin binding	21	2.1	2.40E-04
Protein C-terminus binding	22	2.2	5.00E-04
rRNA binding	9	0.9	8.90E-04
Integrin binding	15	1.5	1.00E-03
Single-stranded DNA-dependent ATPase activity	5	0.5	1.20E-03
Protein kinase binding	34	3.5	2.30E-03
RNA binding	45	4.6	2.70E-03
Calcium ion binding	55	5.6	3.90E-03
P-P-bond-hydrolysis-driven protein transmembrane transporter activity	4	0.4	6.40E-03
DNA clamp loader activity	4	0.4	6.40E-03
Telomerase RNA binding	5	0.5	7.90E-03
Actin filament binding	15	1.5	8.60E-03
Single-stranded DNA binding	12	1.2	8.60E-03
GTPase activator activity	25	2.5	1.10E-02

Table S4: Pathway analysis of genes coexpressed with *PTPN3* (protein tyrosine phosphatase non-receptor type 3) **(**from public lung cancer databases using the MetaCore database (with $p<0.05$ set as the cutoff value)

#	Maps	pValue	Network Objects from Active Data
1	Cell adhesion_Tight junctions	4.779E-05	CGNL1, AF-6, ZO-2, EPB41
2	Cell adhesion_Endothelial cell contacts by junctional mechanisms	2.231E-04	AF-6, ZO-2, MAGI-1(BAIAP1)
3	Neurophysiological process_Kappa-type opioid receptor signaling in the central nervous system	5.407E-04	Adenylate cyclase type VI, IP3 receptor, Adenylate cyclase, p90Rsk
4	Development_Early embryonal hypaxial myogenesis	6.431E-04	PTCH1, Adenylate cyclase, KIF27
5	Prostaglandins and leukotrienes-mediated induction of expression of mucins in normal and asthmatic epithelium	1.002E-03	IP3 receptor, Adenylate cyclase, p90RSK2(RPS6KA3)
6	Neurophysiological process_Receptor-mediated axon growth repulsion	1.220E-03	Ephrin-A, VAV-2, Plexin B1
7	Development_Non-genomic action of Retinoic acid in cell differentiation	2.269E-03	IP3 receptor, p90RSK2(RPS6KA3), p90Rsk
8	Induction of mucin secretion in airway goblet cells by purinergic receptors	2.269E-03	IP3 receptor, MUNC18, MYO5C
9	Neurophysiological process_Constitutive and regulated NMDA receptor trafficking	3.301E-03	IP3 receptor, Adenylate cyclase, SAP102
10	Protein folding_Membrane trafficking and signal transduction of G-alpha (i) heterotrimeric G-protein	3.385E-03	Adenylate cyclase type VI, Rap1GAP1
11	Development_Generation of pancreatic beta-cells from embryonic stem cells (early stages)	4.132E-03	HNF1-beta, SOX9
12	Development_Positive regulation of STK3/4 (Hippo) pathway and negative regulation of YAP/TAZ function	4.236E-03	KIBRA, Adenylate cyclase, ZO-2

13	Cell adhesion_Gap junctions	4.532E-03	Connexin 32, ZO-2
14	Reproduction_Gonadotropin-releasing hormone (GnRH) signaling	4.579E-03	IP3 receptor, Adenylate cyclase, p90Rsk
15	Neurophysiological process_Regulation of intrinsic membrane properties and excitability of cortical pyramidal neurons	4.757E-03	IP3 receptor, Adenylate cyclase, CACNA1D
16	Nociception_Nociceptin receptor signaling	5.126E-03	IP3 receptor, Adenylate cyclase, p90Rsk
17	Development_PTHR1 in bone and cartilage development	5.511E-03	PTCH1, Adenylate cyclase, SOX9
18	Development_MAG, Reticulon 4 and OMgp in inhibition of neurite outgrowth	5.710E-03	SHRM, Rap1GAP1, SH3RF
19	Cell adhesion_Classical cadherin-mediated cell adhesion	6.302E-03	VAV-2, MAGI-1(BAIAP1)
20	Hedgehog signaling in prostate cancer	6.302E-03	PTCH1, KIF27
21	GLP-1 in inhibition of insulin secretion in type 2 diabetes	7.286E-03	Adenylate cyclase type VI, CACNA1D
22	Neurophysiological process_GABA-B receptor signaling in presynaptic nerve terminals	7.286E-03	Adenylate cyclase type VI, MUNC18
23	Tumor-stroma interactions in pancreatic cancer	7.286E-03	PTCH1, KIF27
24	Hedgehog signaling in gastric cancer	7.803E-03	PTCH1, KIF27
25	Inhibition of Ephrin receptors in colorectal cancer	8.336E-03	Ephrin-A, VAV-2
26	Cytoskeleton remodeling_RalA regulation pathway	8.336E-03	REPS2, RalGEF2
27	Development_The role of GDNF ligand family/ RET receptor in cell survival, growth and proliferation	8.695E-03	VAV-2, IP3 receptor, p90RSK2(RPS6KA3)

28	Development_Embryonal epaxial myogenesis	8.885E-03	PTCH1, KIF27
29	Development_Hedgehog signaling	9.221E-03	PTCH1, Adenylate cyclase, SOX9
30	Hedgehog signaling in pancreatic cancer	9.449E-03	PTCH1, KIF27
31	G-protein signaling_Regulation of CDC42 activity	1.003E-02	p200RhoGAP, Zizimin 1
32	Chemotaxis_CCL19- and CCL21-mediated chemotaxis	1.003E-02	IP3 receptor, Adenylate cyclase
33	SHH signaling in melanoma	1.003E-02	PTCH1, KIF27
34	Development_FGF2 signaling during embryonic stem cell differentiation	1.062E-02	PTCH1, SOX9
35	G-protein signaling_RhoA regulation pathway	1.062E-02	Ephrin-A, p200RhoGAP
36	G-protein signaling_S1P2 receptor signaling pathway	1.124E-02	IP3 receptor, Adenylate cyclase
37	G-protein signaling_Regulation of RAC1 activity	1.186E-02	p200RhoGAP, VAV-2
38	Development_Mu-type opioid receptor signaling	1.316E-02	VAV-2, IP3 receptor
39	Signal transduction_Beta-adrenergic receptors signaling via Cyclic AMP	1.316E-02	Adenylate cyclase type VI, Adenylate cyclase
40	Cytoskeleton remodeling_ACM3 and ACM4 in keratinocyte migration	1.316E-02	Adenylate cyclase type VI, IP3 receptor
41	Signal transduction_Cyclic AMP signaling	1.316E-02	KDELR, Adenylate cyclase type VI
42	TrkB signaling in Huntington's disease	1.383E-02	IP3 receptor, p90Rsk

43	Development_PACAP signaling in neural cells	1.383E-02	IP3 receptor, p90RSK2(RPS6KA3)
44	Neurophysiological process_Delta-type opioid receptor in the nervous system	1.451E-02	Adenylate cyclase type VI, IP3 receptor
45	G-protein signaling_Rap1A regulation pathway	1.451E-02	Rap1GAP1, MAGI-1(BAIAP1)
46	Reproduction_Progesterone-mediated oocyte maturation	1.451E-02	Adenylate cyclase, p90Rsk
47	Development_Growth hormone-releasing hormone (GH-RH) signaling	1.521E-02	IP3 receptor, Adenylate cyclase
48	Cytoskeleton remodeling_Role of PKA in cytoskeleton reorganisation	1.521E-02	IP3 receptor, Adenylate cyclase
49	Immune response_PIP3 signaling in B lymphocytes	1.593E-02	IP3 receptor, p90Rsk
50	Transport_ACM3 signaling in salivary glands	1.593E-02	Adenylate cyclase type VI, IP3 receptor

Table S5: Pathway analysis of genes coexpressed with *PTPN5* (protein tyrosine phosphatase non-receptor type 5) from public lung cancer databases using the MetaCore database (with $p<0.05$ set as the cutoff value)

#	Maps	p-value	Network Objects from Active Data
1	Retinal ganglion cell damage in glaucoma	1.224E-05	EDNRB, Factor H, A2M, NGFR(TNFRSF16), TrkC, NSGPeroxidase
2	Development_Transcriptional regulation of megakaryopoiesis	4.779E-05	von Willebrand factor, FLI1, TAL1, CD34, LMO2
3	Development_Growth hormone-releasing hormone (GH-RH) signaling	1.043E-04	CACNA1C, GH-RH, Adenylate cyclase, NAV1.9, GH-RH receptor
4	Development_Endothelial differentiation during embryonic development	2.980E-04	VEGFR-3, SOX18, FOXF1, WNT, TIE2

5	Gamma-secretase regulation of angiogenesis	3.747E-04	NOTCH4 (ICD4), TIE, NOTCH4, TIE2
6	Development_Regulation of endothelial progenitor cell differentiation from adult stem cells	6.375E-04	von Willebrand factor, VE-cadherin, CD34, TIE2, P-selectin
7	Development_Direct reprogramming of cardiac fibroblasts into cardiomyocytes	1.503E-03	Myocardin, TBX5, Alpha-actinin 2
8	Gamma-secretase proteolytic targets	1.860E-03	NOTCH4 (ICD4), NOTCH4, NGFR (ICD), NGFR(TNFRSF16), NGFR (CTF)
9	High shear stress-induced platelet activation	1.933E-03	Alpha-actinin, von Willebrand factor, Gas6, P-selectin
10	TMPRSS2-ERG fusion in Prostate Cancer	1.933E-03	Endoglin, VE-cadherin, ERG, TAL1
11	VEGF signaling in multiple myeloma	2.633E-03	VEGFR-3, MDR1, Caveolin-1, VEGF-D
12	Development_EDNRB signaling	2.633E-03	Endothelin-3, EDNRB, CALDAG-GEFI, Caveolin-1
13	Development_ROBO2, ROBO3 and ROBO4 signaling pathways	2.906E-03	SLIT3, SLIT1, ROBO4
14	Cell adhesion_Endothelial cell contacts by junctional mechanisms	3.257E-03	Alpha-actinin, VE-cadherin, JAM2
15	Platelet activation during ADAM-TS13-deficient thrombotic microangiopathy development	4.035E-03	von Willebrand factor, CALDAG-GEFI, P-selectin
16	Cytoskeleton remodeling_Regulation of actin cytoskeleton organization by the kinase effectors of Rho GTPases	4.517E-03	Alpha-actinin, RhoJ, MyHC, Cdc42 subfamily
17	Development_Regulation of epithelial-to-mesenchymal transition (EMT)	6.413E-03	VE-cadherin, NOTCH4, TCF8, WNT
18	Development_Positive regulation of WNT/Beta-catenin signaling at the receptor level	6.413E-03	R-spondins, R-spondin 1, WNT, Caveolin-1
19	Role of GSK3 beta in cardioprotection against myocardial infarction	6.443E-03	CRLR, RAMP2, RAMP3

20	Development_Role of cell-cell and ECM-cell interactions in oligodendrocyte differentiation and myelination	7.008E-03	Connexin 47, NGFR(TNFRSF16), Neurofascin
21	Development_Angiopoietin - Tie2 signaling	7.600E-03	TIE, Angiopoietin 3, TIE2
22	Neurophysiological process_Thyroliberin in cell hyperpolarization and excitability	8.222E-03	CACNA1C, KCNH2, KCNH7
23	E-cadherin signaling and its regulation in gastric cancer	8.222E-03	Alpha-actinin, TCF8, WNT
24	Role of red blood cell adhesion to endothelium in vaso-occlusion in Sickle cell disease	8.873E-03	von Willebrand factor, DARC, P-selectin
25	Signal transduction_Beta-adrenergic receptors signaling via Cyclic AMP	9.553E-03	CACNA1C, Adenylate cyclase, Phospholemman
26	Defective efferocytosis in COPD	9.553E-03	Gas6, ELMO1, PECAM1
27	Complement pathway disruption in thrombotic microangiopathy	1.026E-02	von Willebrand factor, Factor H, P-selectin
28	Neurophysiological process_Regulation of intrinsic membrane properties and excitability of cortical pyramidal neurons	1.063E-02	CACNA1C, Adenylate cyclase, NAV1.9, ACM1
29	Development_SLIT-ROBO1 signaling	1.100E-02	SLIT3, SLIT1, FLII
30	Breast cancer (general schema)	1.177E-02	DHH, NOTCH4, GHR
31	Development_TGF-beta family mediated differentiation of embryonic stem cells	1.257E-02	SOX17, CD34, PECAM1
32	Role of platelets in allograft rejection	1.340E-02	von Willebrand factor, Kainate receptor, P-selectin
33	Regulation of relaxation of airway smooth muscle cells	1.515E-02	Adenylate cyclase type IV, HSP20, Prostacyclin receptor
34	Muscle contraction_GPCRs in the regulation of smooth muscle tone	1.568E-02	CACNA1C, Adenylate cyclase, Prostacyclin receptor, MyHC

35	Involvement of VEGF signaling in the progression of lung cancer	1.618E-02	VEGFR-3, VEGF-D
36	Development_Schema: FGF signaling in embryonic stem cell self-renewal and differentiation	1.703E-02	SOX17, TAL1, CD34
37	Inhibition of remyelination in multiple sclerosis: role of cell-cell and ECM-cell interactions	1.703E-02	Connexin 47, NGFR(TNFRSF16), Neurofascin
38	Regulation of angiogenesis in prostate cancer	1.703E-02	VEGFR-3, TIE2, VEGF-D
39	Chemokines in inflammation in adipose tissue and liver in obesity, type 2 diabetes and metabolic syndrome X	1.801E-02	CD34, PECAM1, P-selectin
40	Transcription_Assembly of RNA Polymerase II preinitiation complex on TATA-less promoters	1.806E-02	IGFRB, MDR1
41	Development_NOTCH-induced EMT	2.003E-02	VE-cadherin, NOTCH4
42	Ethanol/Acetaldehyde-dependent stimulation of MMP-9 expression in HCC	2.003E-02	ADHG, ADH1
43	Probable BMP4-mediated induction of EMT in airway epithelium	2.423E-02	Desmin, TCF8
44	Gamma-secretase regulation of mammary cell development	2.423E-02	NOTCH4 (ICD4), NOTCH4
45	Blood coagulation_GPVI-dependent platelet activation	2.457E-02	von Willebrand factor, CALDAG-GEFI, PECAM1
46	Immune response_CCL2 signaling	2.457E-02	VE-cadherin, DARC, Caveolin-1
47	Regulation of Adenylate cyclase and IMPA1 by lithium in major depressive disorder	2.646E-02	Adenylate cyclase, P2X2
48	Platelet activation as a result of endothelial dysfunction after stenting	2.701E-02	PDE2A, Adenylate cyclase, Prostacyclin receptor
49	Airway smooth muscle contraction in asthma	2.701E-02	CACNA1C, Adenylate cyclase, MyHC

50	Gamma-Secretase regulation of neuronal cell development and function	2.827E-02	NGFR (ICD), NGFR(TNFRSF16), NGFR (CTF)

Table S6: Pathway analysis of genes coexpressed with *PTPN6* (protein tyrosine phosphatase non-receptor type 6) from public lung cancer databases using the MetaCore database (with $p<0.05$ set as the cutoff value)

#	Maps	p-value	Network Objects from Active Data
1	Immune response_T cell co-signaling receptors, schema	7.148E-19	TIGIT, CD2, SLAM, ICOS, CD86, PP2135, CD40(TNFRSF5), TR2(TNFRSF14), CD48, LAIR1, TIM-3, PD-1, Galectin-9, CD244, CD30(TNFRSF8), CD40L(TNFSF5), MHC class II, OX40(TNFRSF4)
2	Breakdown of CD4+ T cell peripheral tolerance in type 1 diabetes mellitus	6.942E-17	JAK3, CXCR5, Lck, ICOS, CD86, ZAP70, CD3, FOXP3, LAT, CD40(TNFRSF5), STAT5, CD4, HLA-DRB1, PD-1, CD40L(TNFSF5), MHC class II
3	Chemotaxis_SDF-1/ CXCR4-induced chemotaxis of immune cells	9.501E-16	PI3K cat class IA, CD3 zeta, Csk, ITGB2, JAK3, Btk, Lck, Pyk2(FAK2), ZAP70, CD3, VAV-1, alpha-L/beta-2 integrin, CD45, CALDAG-GEFI, SFK, PI3K reg class IB (p101), WASP, PLC-beta
4	COVID-19: immune dysregulation	5.350E-15	Btk, HLA-DRA1, CXCL16, HLA-DPB1, CCR5, HLA-DPA1, Caspase-1, CD3, FOXP3, alpha-L/beta-2 integrin, STAT5, CD4, CCR7, HLA-DMB, HLA-DRB1, ITGAL, TIM-3, PD-1, MHC class II
5	Cell adhesion_Integrin inside-out signaling in T cells	5.447E-15	CD3 zeta, Csk, ITGB2, JAK3, CCL19, URP2, Lck, ZAP70, CD3, VAV-1, alpha-L/beta-2 integrin, CALDAG-GEFI, CCR7, PREL1, PLC-beta2
6	SLE genetic marker-specific pathways in T cells	6.491E-15	CD3 zeta, Csk, RUNX3, Ikaros, MHC class II beta chain, Lck, ZAP70, CD3, VAV-1, LAT, HLA-DRB, Slp76, HLA-DRB1, PD-1, HLA-DRB3, CD40L(TNFSF5), NIK(MAP3K14), MHC class II, OX40(TNFRSF4)
7	Cell adhesion_Integrin inside-out signaling in neutrophils	1.854E-13	ITGB2, Btk, DAP12, Hck, URP2, Fc gamma RI, alpha-L/beta-2 integrin, Slp76, CALDAG-GEFI, PI3K reg class IB (p101), PREX1, FGR, PSGL-1, alpha-M/beta-2 integrin, PREL1, PLC-beta2
8	Immune response_Immunological synapse formation	3.088E-13	PI3K cat class IA, ITGB2, URP2, CD86, ZAP70, CD3, VAV-1, LAT, alpha-L/beta-2 integrin, Slp76, CALDAG-GEFI, WASP, PREL1, MHC class II
9	Rheumatoid arthritis (general schema)	1.651E-12	CD2, MHC class II beta chain, CD86, FOXP3, Fc gamma RI, HLA-DRB, CD40(TNFRSF5), alpha-L/beta-2 integrin, TNF-R2, CD4, HLA-DRB1, CD40L(TNFSF5), MHC class II
10	Maturation and migration of dendritic cells in skin sensitization	2.550E-12	MHC class II alpha chain, HLA-DRA1, CCL19, MHC class II beta chain, CD86, HLA-DRB, CD40(TNFRSF5), TNF-R2, CCR7, HLA-DRB1, HLA-DRB3, MHC class II

11	Immune response_Inhibitory PD-1 signaling in T cells	3.741E-12	PI3K cat class IA, CD3 zeta, Csk, Lck, CD86, ZAP70, CD3, VAV-1, FOXP3, alpha-L/beta-2 integrin, CD4, PD-1, MHC class II
12	Differences between Langerhans cells and dermal dendritic cells in allergic contact dermatitis	4.617E-11	CCL19, ICOS, CD86, CD40(TNFRSF5), CD4, CCR7, PD-1, CD40L(TNFSF5), MHC class II
13	T follicular helper cell dysfunction in SLE	3.771E-10	PI3K cat class IA, JAK3, CXCR5, SLAM, ICOS, CD86, CD40(TNFRSF5), IL-21 receptor, CD4, PD-1, CD40L(TNFSF5), NIK(MAP3K14), MHC class II, OX40(TNFRSF4)
14	Chemokines in inflammation in adipose tissue and liver in obesity, type 2 diabetes and metabolic syndrome X	3.956E-10	ITGAX, ITGAM, CCR5, CD86, CD3, Fc gamma RI, CD68, CD45, ITGAL, PSGL-1, MHC class II
15	Role of Langerin+ dermal dendritic cells in contact hypersensitivity	7.162E-10	ITGAX, ITGB2, CCL19, alpha-X/beta-2 integrin, CCR7, PSGL-1, ITGB7, FLT3 ligand
16	CHDI_Correlations from Replication data_Causal network (positive correlations)	7.913E-10	PI3K cat class IA, Lck, Pyk2(FAK2), ZAP70, CD3, LAT, CD40(TNFRSF5), CD45, Slp76, PI3K reg class IB (p101), MSK1/2 (RPS6KA5/4), NIK(MAP3K14), MHC class II
17	Immune response_NF-AT in immune response	7.969E-10	PI3K cat class IA, CD3 zeta, Btk, Lck, CD86, ZAP70, CD3, VAV-1, LAT, Slp76, MHC class II
18	Renal tubulointerstitial injury in Lupus Nephritis	9.043E-10	CCL19, CXCR5, M-CSF receptor, CCR5, CD40(TNFRSF5), alpha-L/beta-2 integrin, TWEAK(TNFSF12), TNF-R2, CD4, CCR7, CD40L(TNFSF5), MHC class II
19	Immune response_Induction of the antigen presentation machinery by IFN-gamma	1.237E-09	CIITA, NLRC5, HLA-DRA1, HLA-DPB1, HLA-DPA1, CD74, HLA-DMB, HLA-DRB1, PSMB10, MHC class II, HLA-F
20	Role of B cells in SLE	3.428E-09	PI3K cat class IA, Btk, ICOS, CD86, CD40(TNFRSF5), alpha-L/beta-2 integrin, CD45, TNF-beta, CD40L(TNFSF5), MHC class II, OX40(TNFRSF4)
21	Immune response_Differentiation of natural regulatory T cells	5.395E-09	JAK3, CD86, FOXP3, CD40(TNFRSF5), STAT5, CD4, CD40L(TNFSF5), MHC class II, OX40(TNFRSF4)
22	Immune response_IL-2 signaling via JAK/ STAT	5.710E-09	JAK3, IL-2R gamma chain, Cyclin D2, IL-2R beta chain, FOXP3, STAT5A, STAT5, TNF-beta
23	Immune response_M-CSF-receptor signaling pathway	9.304E-09	PI3K cat class IA, PU.1, Cyclin D2, Rac2, DAP12, M-CSF receptor, Hck, Pyk2(FAK2), VAV-1, STAT5A, GAB3, WASP
24	Populations of skin dendritic cells involved in contact hypersensitivity	1.104E-08	ITGAX, ITGAM, CD86, CD40(TNFRSF5), CD45, CCR7, MHC class II
25	B-regulatory cells and tumor cells intercellular interaction	1.689E-08	JAK3, LTB, CD86, CD40(TNFRSF5), TNF-R2, IL-21 receptor, EBI3, PD-1, CD5, CD40L(TNFSF5), OX40(TNFRSF4)

26	Putative role of Tregs in COPD	2.869E-08	CD3 zeta, ZAP70, CD3, FOXP3, LAT, CD45, STAT5, CD4
27	Role of cell adhesion in vaso-occlusion in Sickle cell disease	3.824E-08	ITGB2, ITGAM, CD3, Fc gamma RI, alpha-L/beta-2 integrin, CD45, ITGAL, PSGL-1, alpha-M/beta-2 integrin
28	Immune response_IL-12 signaling pathway	4.944E-08	PI3K cat class IA, CD3 zeta, RUNX3, CXCR5, IL-12RB1, Lck, ICOS, CD3, STAT5, PD-1, CD40L(TNFSF5)
29	G-protein signaling_N-RAS regulation pathway	6.510E-08	CD3 zeta, Lck, ZAP70, CD3, LAT, CALDAG-GEFI, CD4, MHC class II
30	Immune response_TCR alpha/beta signaling pathway	9.866E-08	Csk, Lck, CD86, ZAP70, CD3, VAV-1, LAT, CD45, Slp76, CD4, WASP, MHC class II
31	Immune response_Antigen presentation by MHC class I: cross-presentation	1.241E-07	LLIR, Rac2, DAP12, VAV-1, Fc gamma RI, CD40(TNFRSF5), C1q, CD74, Fc epsilon RI gamma, p47-phox, IP-30, TIM-3
32	CHDI_Correlations from Replication data_Cytoskeleton and adhesion module	1.253E-07	ITGB2, ZAP70, CD3, VAV-1, alpha-L/beta-2 integrin, Slp76, CALDAG-GEFI, PI3K reg class IB (p101), WASP, MHC class II
33	Neutrophil-derived granule proteins and cytokines in asthma	1.274E-07	Rac2, DAP12, IL-9 receptor, Hck, IL9R, Fc epsilon RI gamma, FGR, alpha-M/beta-2 integrin, Alpha-defensin
34	Immune response_CD40 signaling	1.458E-07	PI3K cat class IA, JAK3, Cyclin D2, CD86, CD40(TNFRSF5), TRAF1, STAT5A, TNF-beta, CD40L(TNFSF5), NIK(MAP3K14)
35	Role of keratinocytes and Langerhans cells in skin sensitization	1.504E-07	CARD5, CCL19, Caspase-1, TNF-R2, CCR7, NIK(MAP3K14), MHC class II
36	Inhibition of neutrophil migration by proresolving lipid mediators in COPD	3.438E-07	ITGB2, Rac2, CCR5, VAV-1, alpha-L/beta-2 integrin, TNF-R2, PI3K reg class IB (p101), PREX1, alpha-M/beta-2 integrin, PLC-beta2
37	CCR7 signaling pathways in dendritic cells in allergic contact dermatitis	4.950E-07	CCL19, Rac2, Pyk2(FAK2), alpha-L/beta-2 integrin, CALDAG-GEFI, CCR7, PI3K reg class IB (p101), WASP, PLC-beta2
38	Aberrant production of IL-2 and IL-17 in SLE T cells	5.773E-07	CD3 zeta, Lck, ZAP70, CD3, LAT, CD3 epsilon, Fc epsilon RI gamma, CD4, MHC class II
39	Oxidative stress_Activation of NADPH oxidase	6.711E-07	PI3K cat class IA, Rac2, VAV-1, PI3K reg (p87-gamma), p47-phox, p40-phox, PI3K reg class IB (p101), PREX1, PLC-beta
40	Immune response_ICOS signaling pathway in T-helper cell	7.779E-07	PI3K cat class IA, CXCR5, ICOS, CD3, VAV-1, FOXP3, CD4, CD40L(TNFSF5), MHC class II

41	Differentiation of Th2 cells in asthma	8.462E-07	PI3K cat class IA, CCR5, CD86, CD3, CXCR3, CD4, MHC class II, OX40(TNFRSF4)
42	Immunological synapse between dendritic and CD8+ T cells in allergic contact dermatitis	9.507E-07	CD2, Lck, ICOS, CD86, alpha-L/beta-2 integrin, PD-1, OX40(TNFRSF4)
43	Dual function of Treg cells in cancer development	1.009E-06	ICOS, FOXP3, CD45, STAT5, CD4, TIM-3, PD-1, OX40(TNFRSF4)
44	Immune response_KLRK1 (NKG2D) signaling pathway	1.198E-06	PI3K cat class IA, DAP12, Lck, ZAP70, VAV-1, STAT5, Slp76, DAP10
45	Common mechanisms of Th17 cell migration	1.417E-06	CD43, CXCL16, CCR5, alpha-L/beta-2 integrin, CXCR3, PI3K reg class IB (p101), PSGL-1, PLC-beta
46	NK cells in allergic contact dermatitis	1.476E-06	CIITA, HLA-DRA1, CCR5, alpha-L/beta-2 integrin, CXCR3, STAT5, MHC class II
47	Cell adhesion_Role of tetraspanins in the integrin-mediated cell adhesion	1.957E-06	ITGB2, CD53, alpha-L/beta-2 integrin, CD37, Tspan32, ITGAL, alpha-M/beta-2 integrin, ICAM3
48	G-protein signaling_Regulation of RAC1 activity	2.224E-06	DEF6, Caspase-1, ZAP70, CD3, VAV-1, ARHGAP9, Slp76
49	Immune response_Regulation of T cell function by CTLA-4	2.703E-06	PI3K cat class IA, CD3 zeta, Lck, CD86, ZAP70, CD3, LAT
50	Immune response_Generation of memory CD4+ T cells	2.703E-06	JAK3, CD86, STAT5, CD4, CCR7, MHC class II, OX40(TNFRSF4)

Table S7: Pathway analysis of genes coexpressed with *PTPN13* (protein tyrosine phosphatase non-receptor type 13) from public lung cancer databases using the MetaCore database (with $p<0.05$ set as the cutoff value)

#	Maps	p-value	Network Objects from Active Data
1	Immune response_HMGB1 release from the cell	1.292E-06	JNK(MAPK8-10), PCAF, CRM1, TLR2, PI3K reg class IA (p85), PI3K reg class IA
2	Putative pathways of hormone action in neurofibromatosis type 1	2.662E-06	PI3K reg class IA (p85-alpha), PI3K reg class IA (p85), GHR, PI3K reg class IA, PR (nuclear)

3	Immune response_HMGB1/RAGE signaling pathway	1.173E-04	JNK(MAPK8-10), PI3K reg class IA (p85-alpha), TLR2, MEF2C, PI3K reg class IA (p85)
4	Development_Role of HDAC and calcium/calmodulin-dependent kinase (CaMK) in control of skeletal myogenesis	1.173E-04	MEF2, MAP3K3, PCAF, MEF2C, PI3K reg class IA
5	Immune response_Gastrin in inflammatory response	3.823E-04	JNK(MAPK8-10), MEF2, MEF2C, PKC-epsilon, PI3K reg class IA (p85)
6	Breast cancer (general schema)	5.135E-04	ErbB4, PR (membrane), GHR, PR (nuclear)
7	Development_Neurotrophin family signaling	5.135E-04	GAB1, JNK(MAPK8-10), PI3K reg class IA (p85), PI3K reg class IA
8	Populations of skin dendritic cells involved in contact hypersensitivity	5.465E-04	Langerin, CD1d, CD1a
9	Growth factors in regulation of oligodendrocyte precursor cells survival in multiple sclerosis	5.636E-04	JNK(MAPK8-10), ErbB4, PI3K reg class IA (p85), PI3K reg class IA
10	PR action in breast cancer: stimulation of metastasis	7.534E-04	PI3K reg class IA (p85-alpha), PR (membrane), PR (nuclear)
11	CHDI_Correlations from Replication data_Causal network (positive correlations)	7.633E-04	JNK(MAPK8-10), MEF2, TLR2, HIP1, PI3K reg class IA (p85)
12	Gamma-secretase regulation of mammary cell development	8.732E-04	ErbB4, ErbB4(ICD), ErbB4(CTF)
13	Neutrophil-derived granule proteins and cytokines in asthma	1.016E-03	PRG2, CysLT1 receptor, PKC, IP10
14	Development_Thromboxane A2 signaling pathway	1.096E-03	Adenylate cyclase, PKC, PI3K reg class IA (p85), PI3K reg class IA
15	Immune response_Function of MEF2 in T lymphocytes	1.096E-03	MEF2, MAP3K3, PCAF, MEF2C
16	Signal transduction_S1P4 receptor and S1P5 receptor signaling	1.096E-03	JNK(MAPK8-10), CRMP2, Adenylate cyclase, PI3K reg class IA
17	Development_EGFR signaling via PIP3	1.147E-03	GAB1, JNK(MAPK8-10), PI3K reg class IA

18	Immune response_Fc epsilon RI pathway: Lyn-mediated cytokine production	1.181E-03	JNK(MAPK8-10), Fc epsilon RI beta, PKC, MEF2C, Fc epsilon RI alpha
19	Signal transduction_Additional pathways of NF-kB activation (in the cytoplasm)	1.271E-03	Adenylate cyclase, MAP3K3, PKC-epsilon, PI3K reg class IA
20	G-protein signaling_Proinsulin C-peptide signaling	1.271E-03	PI3K reg class IA (p85-alpha), PKC-epsilon, PI3K reg class IA (p85), PI3K reg class IA
21	Development_Endothelin-1/EDNRA signaling	1.271E-03	JNK(MAPK8-10), Adenylate cyclase, PKC-epsilon, PI3K reg class IA
22	Regulation of AKT(PKB)/ GSK3 beta cascade in bipolar disorder	1.464E-03	GAB1, ErbB4, GFRalpha1, PI3K reg class IA (p85)
23	T cell generation in COPD	1.470E-03	Langerin, CD1a, CD1c
24	Immune response_IFN-gamma signaling via PI3K and NF-kB	1.677E-03	PI3K reg class IA (p85-alpha), CX3CL1, PKC-epsilon, IP10
25	G-protein signaling_Rap2B regulation pathway	1.682E-03	MR-GEF, PDZ-GEF1
26	Immune response_LTBR1 signaling	2.165E-03	JNK(MAPK8-10), Adenylate cyclase, CRM1, PKC
27	Role of Neuregulin 1 and Thymosin beta-4 in myocardium regeneration after infarction	2.275E-03	GAB1, ErbB4, PI3K reg class IA (p85)
28	Immune response_Fc epsilon RI pathway: signaling through Fyn and PI3K	2.301E-03	LAMP3, Fc epsilon RI beta, PI3K reg class IA (p85), Fc epsilon RI alpha
29	Development_Gastrin in cell growth and proliferation	2.442E-03	JNK(MAPK8-10), PI3K reg class IA (p85-alpha), PKC-epsilon, PI3K reg class IA (p85)
30	Signal transduction_Adenosine A1 receptor signaling pathway	2.442E-03	Adenylate cyclase, PKC, PKC-epsilon, PI3K reg class IA (p85)
31	Inhibition of mast cell functions by Siglecs in asthma	2.511E-03	Fc epsilon RI beta, Siglec-6, Fc epsilon RI alpha
32	Neurogenesis_NGF/ TrkA MAPK-mediated signaling	2.712E-03	RASGRF1, SORBS1, PDZ-GEF1, MEF2C, PKC-epsilon

33	MAPK-independent proliferation of normal and asthmatic smooth muscle cells	2.743E-03	PI3K reg class IA (p85-alpha), CysLT1 receptor, PI3K reg class IA (p85), PI3K reg class IA
34	TNF-alpha and IL-1 beta-mediated regulation of contraction and secretion of inflammatory factors in normal and asthmatic airway smooth muscle	2.903E-03	JNK(MAPK8-10), JNK3(MAPK10), PCAF, PI3K reg class IA (p85)
35	NF-AT signaling in cardiac hypertrophy	2.903E-03	GAB1, MEF2C, PKC-epsilon, PI3K reg class IA
36	Development_Growth factors in regulation of oligodendrocyte progenitor cell proliferation	3.241E-03	ErbB4, PKC, PI3K reg class IA (p85), PI3K reg class IA
37	G-protein signaling_Regulation of CDC42 activity	3.308E-03	CDGAP, Zizimin 1, RHG7
38	Androgen receptor activation and downstream signaling in Prostate cancer	3.316E-03	GAB1, SPRY2, TMPRSS2, TLR2, ER81
39	Production of reactive oxygen species and arachidonic acid metabolites by neutrophils in asthma	3.419E-03	PRG2, CysLT1 receptor, PKC, PI3K reg class IA (p85)
40	Nicotine / Beta-adrenergic signaling in lung cancer	3.604E-03	Adenylate cyclase, Beta-2 adrenergic receptor, Beta-adrenergic receptor
41	Development_Lipoxin inhibitory action on PDGF, EGF and LTD4 signaling	4.244E-03	Adenylate cyclase, CysLT1 receptor, PI3K reg class IA (p85)
42	Immune response_Antigen presentation by MHC class II	4.476E-03	Langerin, JNK(MAPK8-10), CLEC10A, TLR2, PKC
43	Development_Growth factors in regulation of oligodendrocyte precursor cell survival	4.588E-03	ErbB4, PI3K reg class IA (p85), PI3K reg class IA
44	G-protein signaling_H-RAS regulation pathway	4.588E-03	RASGRF1, GFRalpha1, PDZ-GEF1
45	G-protein signaling_Regulation of p38 and JNK signaling mediated by G-proteins	4.588E-03	JNK(MAPK8-10), MEF2C, PKC-epsilon
46	Development_Beta-adrenergic receptors transactivation of EGFR	4.588E-03	GAB1, Beta-2 adrenergic receptor, PI3K reg class IA
47	G-protein signaling_G-Protein alpha-12 signaling pathway	4.949E-03	JNK(MAPK8-10), MR-GEF, PI3K reg class IA (p85)

48	Apoptosis and survival_Anti-apoptotic action of membrane-bound ESR1	4.949E-03	JNK(MAPK8-10), PKC-epsilon, PI3K reg class IA (p85)
49	Development_Gastrin in differentiation of the gastric mucosa	4.949E-03	VMAT2, PKC, PKC-epsilon
50	HGF signaling in melanoma	5.326E-03	GAB1, JNK(MAPK8-10), PI3K reg class IA (p85)

Table S8: Pathway analysis of genes coexpressed with *PTPN21* (protein tyrosine phosphatase non-receptor type 21) from public lung cancer databases using the MetaCore database (with $p<0.05$ set as the cutoff value)

#	Maps	p-value	Network Objects from Active Data
1	Signal transduction_IGF-1 receptor signaling pathway	7.810E-06	GAB1, JAK1, H-Ras, FKHR, SREBP1 (nuclear), FOXO3A, PI3K reg class IA (p85)
2	Cytoskeleton remodeling_Regulation of actin cytoskeleton organization by the kinase effectors of Rho GTPases	7.810E-06	RhoJ, Vinculin, Spectrin, MLCK, Alpha adducin, MyHC, Cdc42 subfamily
3	Breast cancer (general schema)	1.145E-05	PTHR1, PTCH1, PR (membrane), GHR, PR (nuclear), TGF-beta receptor type II
4	Development_ROBO2, ROBO3 and ROBO4 signaling pathways	1.300E-05	SLIT3, SLIT1, ROBO4, SLIT2, ROBO2
5	Putative pathways of hormone action in neurofibromatosis type 1	1.300E-05	PI3K reg class IA (p85-alpha), PI3K reg class IA (p85), GHR, PI3K reg class IA, PR (nuclear)
6	Development_Role of IL-8 in angiogenesis	1.680E-05	S1P, SREBP1 precursor, PI3K reg class IA (p85-alpha), SREBP1 (nuclear), SREBP1 (Golgi membrane), PI3K reg class IA (p85), Caveolin-1
7	Insulin-dependent stimulation of SREBP-1 in type 2 diabetes in liver	1.936E-05	S1P, SREBP1 precursor, PI3K reg class IA (p85-alpha), SREBP1 (nuclear), SREBP1 (Golgi membrane)
8	Development_Endothelial differentiation during embryonic development	4.133E-05	Angiopoietin 1, FKHR, COUP-TFII, FOXF1, PI3K reg class IA, TIE2

9	Stem cells_Cooperation between Hedgehog, IGF-2 and HGF signaling pathways in medulloblastoma stem cells	4.589E-05	GAB1, PTCH1, TCF7L2 (TCF4), Tcf(Lef), PI3K reg class IA
10	Role of ER stress in obesity and type 2 diabetes	5.746E-05	S1P, SREBP1 precursor, PI3K reg class IA (p85-alpha), SREBP1 (nuclear), SREBP1 (Golgi membrane), PI3K reg class IA (p85)
11	Regulation of lipid metabolism_Regulation of fatty acid synthase activity in hepatocytes	8.156E-05	S1P, SREBP1 precursor, SREBP1 (nuclear), SREBP1 (Golgi membrane)
12	Regulation of lipid metabolism_Regulation of lipid metabolism via LXR, NF-Y and SREBP	1.076E-04	S1P, SREBP1 precursor, SREBP1 (nuclear), SREBP1 (Golgi membrane), Caveolin-1
13	Transcription_Sirtuin6 regulation and functions	1.377E-04	S1P, SREBP1 precursor, FKHR, SREBP1 (nuclear), SREBP1 (Golgi membrane), FOXO3A
14	Main growth factor signaling cascades in multiple myeloma cells	1.558E-04	H-Ras, FKHR, PI3K reg class IA (p85-alpha), PI3K reg class IA (p85), PI3K reg class IA
15	Cell adhesion_PLAU signaling	1.639E-04	JAK1, H-Ras, PI3K reg class IA (p85-alpha), MLCK, MYLK1, Caveolin-1
16	Hypertrophy of asthmatic airway smooth muscle cells	2.471E-04	MYH11, JAK1, Smooth muscle myosin, EDNRB, MLCK, TGF-beta receptor type II
17	Development_Stimulation of differentiation of mouse embryonic fibroblasts into adipocytes by extracellular factors	2.671E-04	H-Ras, SREBP1 precursor, FKHR, Prostacyclin receptor, BMP receptor 2, PI3K reg class IA (p85)
18	High shear stress-induced platelet activation	2.710E-04	von Willebrand factor, PI3K reg class IA (p85-alpha), Vinculin, UFO, PI3K reg class IA (p85)
19	Immune response_IL-6 signaling pathway via MEK/ERK and PI3K/AKT cascades	3.350E-04	GAB1, JAK1, H-Ras, FKHR, PI3K reg class IA (p85), PI3K reg class IA
20	Inhibition of TGF-beta 1 signaling in early colorectal cancer	3.414E-04	Ski, Beta-fodrin, PI3K reg class IA, TGF-beta receptor type II
21	HBV-dependent NF-kB and PI3K/AKT pathways leading to HCC	4.022E-04	JAK1, H-Ras, PI3K reg class IA (p85-alpha), A2M, PI3K reg class IA (p85)
22	IL-6 signaling in breast cancer cells	5.284E-04	GAB1, JAK1, H-Ras, IP10, PI3K reg class IA
23	Development_Role of growth factors in the maintenance of embryonic stem cell pluripotency	5.284E-04	GAB1, H-Ras, PI3K reg class IA (p85), PI3K reg class IA, TGF-beta receptor type II

24	Development_Role of HDAC and calcium/calmodulin-dependent kinase (CaMK) in control of skeletal myogenesis	5.284E-04	CACNA1C, MEF2, MAP3K3, MEF2A, PI3K reg class IA
25	Inhibition of TGF-beta signaling in lung cancer	5.885E-04	Ski, Beta-fodrin, TGF-beta receptor type III (betaglycan), TGF-beta receptor type II
26	Putative pathways for stimulation of fat cell differentiation by Bisphenol A	6.661E-04	SREBP1 precursor, FKHR, TCF7L2 (TCF4), PI3K reg class IA
27	Immune response_IL-15 signaling via MAPK and PI3K cascades	6.823E-04	JAK1, H-Ras, FKHR, FOXO3A, PI3K reg class IA (p85)
28	Mitogenic action of ErbB2 in breast cancer	6.823E-04	H-Ras, FKHR, TCF7L2 (TCF4), FOXO3A, PI3K reg class IA (p85)
29	Development_Cytokine-mediated regulation of megakaryopoiesis	7.404E-04	GAB1, JAK1, FOXO3A, PI3K reg class IA (p85), PI3K reg class IA
30	Regulation of lipid metabolism_Insulin regulation of fatty acid metabolism	8.999E-04	S1P, H-Ras, SREBP1 precursor, SREBP1 (nuclear), SREBP1 (Golgi membrane), PI3K reg class IA
31	Development_Regulation of endothelial progenitor cell differentiation from adult stem cells	9.369E-04	Angiopoietin 1, von Willebrand factor, CD34, PI3K reg class IA (p85), TIE2
32	Development_Angiopoietin - Tie2 signaling	9.418E-04	Angiopoietin 1, FKHR, PI3K reg class IA, TIE2
33	PR action in breast cancer: stimulation of cell growth and proliferation	1.049E-03	JAK1, H-Ras, PR (membrane), PR (nuclear)
34	CHDI_Correlations from Discovery data_Causal network (positive)	1.049E-03	JAK1, Vinculin, PI3K reg class IA (p85), TIE2
35	Immune response_IL-7 signaling in T lymphocytes	1.165E-03	JAK1, FKHR, PI3K reg class IA (p85-alpha), FOXO3A
36	G-protein signaling_H-RAS regulation pathway	1.165E-03	Angiopoietin 1, H-Ras, PDZ-GEF1, TIE2
37	Development_Beta-adrenergic receptors transactivation of EGFR	1.165E-03	GAB1, H-Ras, MYLK1, PI3K reg class IA
38	Activation of Notch signaling in breast cancer	1.423E-03	H-Ras, HES1, MAML2, TCF8

39	Development_Thrombopoietin signaling via ERK1/2 and PI3K	1.544E-03	GAB1, H-Ras, FOXO3A, TAL1, PI3K reg class IA (p85)
40	WNT signaling in HCC	1.565E-03	TBX3, TCF7L2 (TCF4), Tcf(Lef), Prickle-1
41	Development_FGF2-induced self-renewal of adult neural stem cells	1.565E-03	GAB1, H-Ras, HES1, PI3K reg class IA
42	Development_SLIT-ROBO1 signaling	1.565E-03	SLIT3, SLIT1, SLIT2, PI3K reg class IA (p85)
43	Development_Astrocyte differentiation from adult stem cells	1.565E-03	JAK1, H-Ras, HES1, BMP receptor 2
44	IL-6 signaling in Prostate Cancer	1.565E-03	GAB1, JAK1, H-Ras, PI3K reg class IA
45	Cytoskeleton remodeling_Alpha-1A adrenergic receptor-dependent inhibition of PI3K	1.646E-03	CACNA1C, PI3K reg class IA (p85-alpha), MLCK
46	Development_Growth hormone-releasing hormone (GH-RH) signaling	1.718E-03	CACNA1C, GH-RH, H-Ras, GH-RH receptor
47	Regulation of metabolism_Bile acids regulation of glucose and lipid metabolism via FXR	1.718E-03	SREBP1 precursor, FKHR, SREBP1 (nuclear), FXR
48	Cytoskeleton remodeling_Role of PKA in cytoskeleton reorganisation	1.718E-03	MLCK, Fodrin (spectrin), Alpha adducin, LBC
49	Development_Neurotrophin family signaling	1.718E-03	GAB1, H-Ras, PI3K reg class IA (p85), PI3K reg class IA
50	Stellate cells activation and liver fibrosis	1.877E-03	PTCH1, H-Ras, Tcf(Lef), PI3K reg class IA (p85), TGF-beta receptor type II
